# Non-Alcoholic Wines: Sensory Pleasantness and Health Benefits

**DOI:** 10.3390/foods14081356

**Published:** 2025-04-15

**Authors:** Sílvia Afonso, Ana Luísa Teixeira, Elza Escobar, António Inês, Alice Vilela

**Affiliations:** 1 Centre for the Research and Technology of Agroenvironmental and Biological Sciences (CITAB), Institute for Innovation, Capacity Building and Sustainability of Agri-Food Production (Inov4Agro), University of Trás-os-Montes and Alto Douro (UTAD), Quinta de Prados, 5000-801 Vila Real, Portugal; safonso@utad.pt; 2 University of Trás-os-Montes and Alto Douro (UTAD), 5001-801 Vila Real, Portugal; al72964@alunos.utad.pt (A.L.T.); al67858@alunos.utad.pt (E.E.); 3 Chemistry Research Centre-Vila Real (CQ-VR), Department of Biology and Environment, School of Life Sciences and Environment, University of Trás-os-Montes and Alto Douro (UTAD), 5001-801 Vila Real, Portugal; aines@utad.pt; 4 Chemistry Research Centre-Vila Real (CQ-VR), Department of Agronomy, School of Agrarian and Veterinary Sciences, University of Trás-os-Montes and Alto Douro (UTAD), 5001-801 Vila Real, Portugal

**Keywords:** dealcoholization methods, biodehalcoolization, consumer perception, human health, food pairing

## Abstract

Non-alcoholic wine is becoming popular as a healthier alternative to traditional wine, offering potential health benefits without the risks of alcohol consumption. Sensory attributes, such as taste and aroma, significantly influence consumer preferences, with sweet, sour, and balanced fragrances favored over bitter or medicinal notes. A lower alcohol content can enhance the complexity of sensory properties, suggesting that non-alcoholic wines provide an appealing experience. Moderate consumption, particularly of red wine, has been linked to reduced cardiovascular mortality, attributed to phenolic compounds like resveratrol and quercetin present in both alcoholic and non-alcoholic wines. These bioactive components are associated with reduced risks of chronic diseases by modulating biochemical pathways and gene expression. Health-conscious consumers are increasingly taking these benefits into account in their purchasing decisions. Non-alcoholic wines may appeal to individuals seeking health benefits without the presence of alcohol. While some evidence supports the health advantages of wine, most research is observational, and the specific benefits of non-alcoholic options need further investigation. Challenges include isolating the effects of wine’s bioactive compounds from other factors and creating appealing non-alcoholic wines through innovative fermentation techniques, such as using non-*Saccharomyces* yeast strains. Overall, non-alcoholic wine holds promise for those seeking sensory and health benefits without alcohol, highlighting the need for ongoing research and innovation in production methods to enhance its appeal and validate its benefits. Based on recent findings, this review will examine the sensory qualities and health benefits of non-alcoholic wine.

## 1. Introduction

The trend towards low-alcohol and non-alcoholic beverages is gaining momentum, particularly in higher-income countries. This shift is driven by a desire for healthier lifestyles and a greater variety of beverage options. This shift is part of a broader movement towards premiumization, where consumers prioritize quality over quantity.

A growing interest in health and wellness increases demand for low-alcohol and non-alcoholic beverages. This trend is particularly evident in affluent countries where consumers seek healthier lifestyles [1,2].

The low-alcohol beverage market is expanding beyond beer to include wine and spirits. Australian brewers are recognized for leading the way in developing low- and no-alcohol beer categories, contributing to a reduction in national alcohol consumption [1]. The craft beverage revolution is contributing to the popularity of low-alcohol options, with a focus on taste and quality. Craft breweries in the United States increasingly offer low-alcohol beers, with ales being the most common type [2].

Thus, low-alcohol wines and beers are gaining acceptance as they offer potential health benefits and reduce alcohol-related harm. However, increased awareness and marketing efforts are necessary to boost their popularity further [3]. In Germany, there has been a significant increase in the consumption of non-alcoholic beverages, including juice, soft drinks, and water, over the past two decades, reflecting a shift towards healthier beverage choices [4]. In contrast, in the United States, a decrease has occurred. Interestingly, in the United States, there has been a notable decrease in sugar-sweetened beverages among children, accompanied by a corresponding shift toward low- or no-calorie drinks. However, the consumption of sports and energy drinks has increased slightly [5]. Canadians have reported a decrease in the consumption of sugary drinks and an increase in the consumption of plain water, indicating a positive shift towards healthier beverage choices [6].

### Definition of Low- (Or No)-Alcohol Wines and Legislation

Low- and no-alcohol wines are defined by their reduced alcohol content compared to standard wines. These products are gaining popularity due to health, economic, and social factors and consumer interest in healthier lifestyles [1,7,8].

Legislation surrounding the production of low-alcohol beverages is crucial for ensuring consumer safety and proper market classification. The International Organisation of Vine and Wine (OIV) provides specific definitions and classifications for wine categories, including “low-alcohol” and “non-alcoholic wines”, based on their alcohol content: low-alcohol wine (Vin à Teneur Réduite en Alcool/low-alcohol wine) is a product derived from wine by “partially removing alcohol”, so that the final product still contains “some alcohol” but “less than traditional wine”, between 0.5% and 8.5% (*v*/*v*) (depending on the type and national regulations) [9].

However, there is significant variation and confusion in the legal definitions and regulations of low- and no-alcohol products across different regions. Regulatory Definitions and Compliance: The definitions of terms like “no”, “low”, “zero”, and “reduced” alcohol products lack global harmonization. This inconsistency complicates the classification and labeling of these beverages, highlighting the need for standardized global definitions, possibly at the Codex Alimentarius level [8]. Nevertheless, some classifications are used currently: (i) Low-alcohol wines typically contain between 0.5% and 10.5% (*v*/*v*) alcohol, with specific categories such as dealcoholized (<0.5%, *v*/*v*), low alcohol (0.5–1.2%, *v*/*v*), reduced alcohol (1.2–6.5%, *v*/*v*), and lower alcohol (5.5–10.5%, *v*/*v*) [10]. (ii) No-alcohol wines, often referred to as “alcohol-free”, generally contain less than 0.5% (*v*/*v*) of ethanol [8,10].

**Figure 1:**
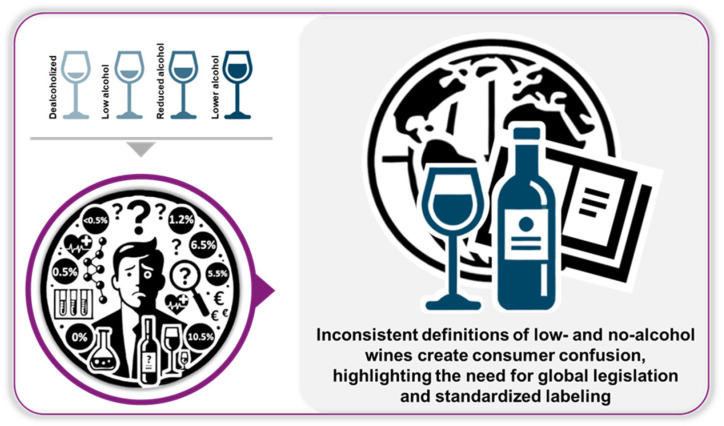
Reduced-alcohol wine classification: challenges in standardization and labeling.

Significant variations exist in the legal definitions and labeling of low- and no-alcohol wines across different regions. This inconsistency has led to confusion among consumers and highlights the need for global harmonization of definitions and labeling standards (Figure 1) [8,11].

According to the OIV [9], commercialized products must be clearly labeled to indicate they are dealcoholized or partially dealcoholized. Labeling may also need to specify the “exact alcohol content”, especially if it is between 0.5% and 1.2% (*v*/*v*), as it is no longer considered entirely “non-alcoholic” under many national laws). National laws may impose stricter standards or use terms such as “alcohol-free”, “light wine”, or “low-alcohol content”. Nevertheless, for instance, in Australia, recent regulatory changes allow wines with as little as 4.5% (*v*/*v*) to be classified as “wine”, aligning more closely with European standards [10], while currently in the UK and EU, legislation limits low-strength verbal descriptors to products with 1.2% (*v*/*v*) or lower. However, there is interest in extending these limits [12].

Accurate measurement of alcohol content is essential for compliance with regulations. Analytical techniques with high sensitivity are necessary to ensure products meet the defined alcohol content levels. Some products have been found to contain more alcohol than the labeled content, highlighting the need for enhanced industry controls and regulatory oversight.

## 2. Dealcoholization Techniques

**Figure 2:**
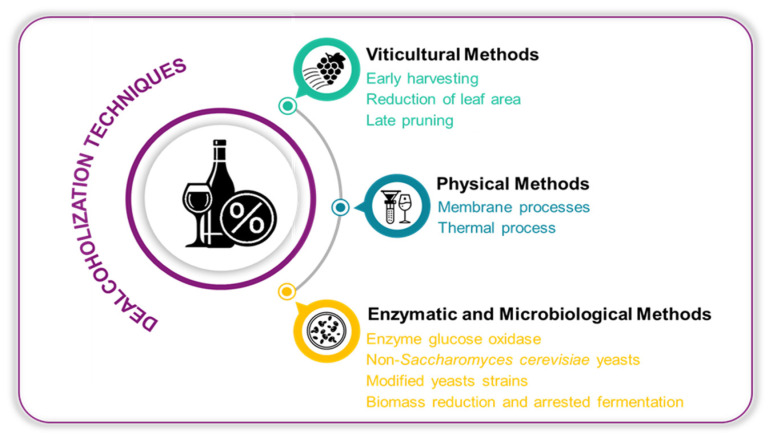
Methods for reducing alcohol content in wines.

Several methods can be employed for wine dealcoholization, with those discussed in this review summarized in Figure 2.

### 2.1. Viticultural Methods

Viticultural techniques implemented in the vineyard aim to limit sugar accumulation in grapes prior to fermentation, thus lowering the final ethanol concentration. In Table 1, an overview of these strategies applied to reduce the alcohol content in wine is presented.

#### 2.1.1. Early Harvesting of Grapes

Determining the harvest date can be a strategy to reduce the final alcohol content in wine, applicable to red and white grape varieties. This approach can include harvesting grapes earlier than usual or blending ripe grapes with early-harvested grapes [13,14]. One specific method called double harvesting involves precisely timing two separate harvests, determining the ideal amount of grapes for each, and choosing the best way to preserve the harvested grapes, either as a must or wine. This method requires experimental adjustments to optimize variables [15]. However, early harvesting can result in wines that have not fully developed their organoleptic properties, as the grapes may not have had enough time to accumulate all the typical flavor precursors before harvest or even present organoleptic defects, such as herbaceous flavors and higher acidity levels than usual [16]. Even so, reduced ethanol concentration in wine can be as high as 3% (*v*/*v*) [17]. Studies using Pinot Noir and Tannat varieties have shown a reduction in alcohol content, accompanied by a decrease in pH and total acidity, without any further effects on other wine constituents [16]. A reduction in alcohol content was also observed for Barbera and Pinot Noir wines, particularly for Barbera grapes, which, when harvested seven days earlier than fully ripe, resulted in wines with a lower alcohol content of 2% (*v*/*v*) [14].

#### 2.1.2. Reduction in Leaf Area

Reducing the leaf area is an effective strategy for managing the alcohol content of wine, as it influences the accumulation of sugars in grapes. Leaves are essential for photosynthesis, a process that converts sunlight into sugars in vineyards, and these sugars are transformed into alcohol by yeast during fermentation. Therefore, the sugar content of the grapes at the time of harvest is decisive for the alcoholic potential of the wine [18]. An adequate ratio between leaf area and fruit, typically between 0.8 and 1.2 m^2^/kg, is crucial for achieving optimal sugar levels in grapes while preserving the wine’s flavor and phenolic content. By strategically pruning or trimming leaves, winemakers can control photosynthesis and, thus, the sugar levels in the grapes, resulting in a lower alcohol content. Winemakers must manage this process to slow sugar accumulation at specific stages of grape development without compromising other essential characteristics such as flavor, tannins, and acidity [19,20,21]. The effectiveness of this technique can vary depending on variety and environmental conditions, highlighting the importance of winegrowers understanding the particularities of their vineyards and the impact of canopy management practices [22]. Studies with Syrah have shown that apical defoliation, which involves removing the leaves from the top of the vine, can slow sugar accumulation and reduce the final alcohol content of the wine without compromising the aromatic qualities [23]. Similarly, removing leaves after veraison effectively reduces sugar content in Sangiovese grapes, resulting in wines with a lower alcohol content while maintaining flavor profiles [24]. However, these results are inconsistent across all varieties, as observed for Corot Noir [25] and Cabernet Sauvignon [26], where bud thinning increased both the soluble solids and alcohol content of wines, suggesting a complex interaction between agronomical practices and biochemical processes [27]. This emphasizes the need for an adapted approach to canopy management, tailored to the characteristics and needs of each variety, to achieve the desired wine profiles. The effectiveness of practices such as bud thinning and leaf area reduction varies significantly depending on the array, so careful planning and precise implementation of these techniques are essential to influence the alcohol content, flavor profile, and overall quality of the final wine [28].

Defoliation can influence several aromatic molecules in grape cluster areas, including volatile compounds, glycosylated aroma precursors, C13-norisoprenoids, methoxy-pyrazines, and terpenes [29]. For example, removing five basal leaves from each shoot at veraison in the Riesling Italico and Traminer grape varieties significantly increased the flavonoid content of the grapes. In contrast, untreated vines generally contained lower levels of hydroxybenzoic acid, catechin, and epicatechin [30]. Sun exposure positively impacted aromatic compounds, such as linalool and geraniol [31]. In the Isonzo region of Italy, increased sun exposure early in berry development reduced the concentrations of certain methoxypyrazines, although no significant differences were observed at harvest time. Furthermore, removing leaves two weeks before veraison increased the monoterpenes in Sauvignon Blanc grapes [32]. In Muscat grapes, shaded berries had higher levels of volatile compounds such as hexanal, while terpenols were lower [33]. In Istrian Malvasia vines, both manual and mechanical leaf removal increased the composition of hydroxynamates and aromatic thiol precursors in the berries, improving the aroma and flavor. These variations can be attributed to differences in bunch temperature and phytochrome activity, influencing monoterpene biosynthesis [34].

#### 2.1.3. Late Pruning

Several studies have found that postponing the dry pruning period (winter) can delay grape ripening [35,36]. The proposed techniques involve winter pruning that is delayed by 1 to 3 months, approximately the usual date of effect, occurring in the vegetative phase after the budding of the following season. This delayed pruning allows the entire vegetative–productive cycle to advance in time, with more evident results in short pruning systems rather than in long ones. In this way, it is possible to obtain considerable reductions in sugar concentration, less degradation of organic acids, and optimal pH values of the grape-must without negative influences on the anthocyanin and phenolic content [37]. A reasonable application appears to be in agricultural short pruning systems, which consist of mechanized pre-pruning performed in mid-winter to a height of 8 to 10 buds, followed by a spring trim to select and shorten the buds. This approach has been shown to delay berry ripening and reduce sugar accumulation in grapes. This can lead to wines with lower alcohol content and improved phenolic profiles, as seen in studies on Merlot and Sangiovese grapevines [19,38,39]. Short canopy management and late pruning have been shown to reduce wine alcohol concentration by decreasing the berry total soluble solids (TSSs) at harvest. These practices also enhance the sensory attributes of wine, such as phenolic content and acidity, which are crucial for wine quality [19,20,40].

However, the effectiveness of pruning techniques can vary significantly between grape varieties. For instance, late pruning has a more pronounced effect on Syrah than on Malbec, indicating the need for varietal-specific strategies [41]. While these pruning techniques can improve wine quality, they often reduce yields, impacting economic viability. Further research is needed to optimize these practices, balancing quality improvements and sustainable production levels [38,39].

### 2.2. Physical Methods

A summary of the primary physical methods employed for wine dealcoholization is presented in Table 2. These techniques, applied after fermentation, involve membrane- or heat-based processes to remove ethanol while retaining desirable sensory attributes selectively.

#### 2.2.1. Membrane Dealcoholization Processes

Membrane separation processes have been achieving increasing success, presenting numerous advantages that allow them to compete with classical separation techniques and thermal processes [18,42]. The most used membrane separation techniques include pervaporation (PV), osmotic distillation (OD), nanofiltration, and reverse osmosis (RO). Except for the pervaporation and osmotic distillation techniques, most dealcoholization processes using membranes promote separation without a phase change, justifying the energy savings. In these processes, the selectivity of the materials to be separated is very high, typically at room temperature. The execution and expansion of these processes are straightforward, enabling the application of data obtained from a pilot scale to an industrial scale [43]. Combining different membranes is of great interest, as it allows the advantages of one to be enjoyed while mitigating some of the disadvantages of another [44]. One of these processes is pervaporation, which involves a concentration gradient. It is a membrane separation technique that utilizes membranes composed of hydrophobic materials, which bind preferentially to organic compounds—those with nonpolar properties—rather than inorganic compounds—those with polar properties. The selective permeable and non-porous membrane triggers partial vaporization of the liquid, resulting in a permeate in a vapor state and a portion retained in a liquid state [45]. The main driving force of pervaporation separation is the function of membrane material and the interaction of the type of liquid (wine, beer) to be removed [46]. Pervaporation, particularly for reducing alcohol in wine, is viable at low temperatures and pressures. This practice may result in a higher alcohol concentration on the permeate side, thereby reducing the density of feed solutions [45,46,47]. In this process, in which substances cross the membrane and change from a liquid to a vapor state, most of the aroma compounds are concentrated in the permeate, with an alcohol content of 40 mL per 100 mL and, consequently, the retained product can be described as “wine” without alcohol and aroma [48]. In tests carried out by Takács et al. [45] using white wine with 13.11% alcohol (*v*/*v*), it was demonstrated that during the dealcoholization of wine up to 0.5% vol., approximately 70% of the aroma compounds are lost. The resulting permeate had a high content of aroma compounds and a 35 to 38% (*v*/*v*) alcohol concentration. These losses are intolerable and are reflected in the quality of the wine.

Another membrane separation technique is osmotic distillation, a technology in which an aqueous phase containing volatile compounds circulates through hydrophobic membranes in the form of “hollow fibers” and a second aqueous phase, called the carrier liquid (“shipping”) that flows on the opposite side of the membranes [49]. In reducing the alcohol content of a wine, the carrier liquid consists of oxygen-free water. It is pumped through the membrane as a carrier agent (“shipping agent”) in a circuit opposite to the wine (feed). Degassed water enables the transfer of ethanol without displacing water across the membrane. Ethanol evaporates in the feed and is transported by vapor diffusion through the membrane pores to the carrier liquid. The membrane functions as a “vapor gap” between two aqueous phases, in which any volatile compound is free to migrate, either by convection or diffusion [18,50]. Ethanol has a relatively high vapor pressure, making it one of the most volatile compounds in wine, and transfers through the membrane pores are faster than with water and other major wine components. The vapor pressure of aromatic compounds is low, resulting in low flow rates in osmotic distillation. Furthermore, the solubility of aromatic compounds in wine is more excellent in ethanol than in pure water. Diban et al. [50] demonstrated that this process decreases the concentration of most aromatic compounds in the final product. Compounds with high hydrophobicity exhibit adsorption on the contact membrane. These losses increase with the retention time, approaching almost total disappearance. The same team states that this is a credible technology for the partial dealcoholization of 2% (*v*/*v*) without a considerable change in the quality of the resulting wines. On the other hand, Bes [51] found that reducing the alcohol content by 2% (*v*/*v*) through osmotic distillation yields wines that are significantly different from the initial wine.

Nanofiltration (NF) utilizes a semipermeable membrane with a pore size ranging from 1 to 10 nm, enabling it to reject smaller molecules, such as sugars and proteins, at a pressure of approximately 75 bar, surpassing the performance of ultrafiltration membranes [52]. A study has shown that nanofiltration (NF) can reduce the alcohol content in Egri Cuvée vintage wine from 12.0% (*v*/*v*) to between 6.0% and 4.0% (*v*/*v*). This process not only preserves the organoleptic properties of the wine with minimal loss of anthocyanins at lower pressures, making it more cost-effective, but it also significantly enhances the phenolic composition [53].

In contrast, the reverse osmosis (RO) process utilizes high pressures of 60 to 80 bar to force water and ethanol molecules through a semi-permeable membrane, leaving behind a retentate that contains other compounds. This generates a permeate flow enriched in water and ethanol. However, to achieve efficient dealcoholization, water must be added to the retentate, which poses a disadvantage due to legal restrictions in many wine-producing countries [54]. Red wines, such as Cabernet Sauvignon, treated with RO have demonstrated that this membrane filtration process also yields a higher phenolic content than the initial wine, with no visible change in the wine’s color [55].

#### 2.2.2. Thermal Process

Another approach uses thermal processes, such as spinning cone columns (SCCs) and vacuum distillation. SCC is a technique that removes volatile compounds, such as aromatic compounds, SO_2_ (sulfur dioxide), or alcohol [18,56]. This method consists of a vertical stainless-steel cylinder in which an inert carrier gas removes, under vacuum, a vapor stream of volatile compounds from liquids or suspensions. Initially, most of the volatile compounds in the wine are removed, followed by alcohol [57,58]. This method can attenuate the alcohol content in wine, achieving the desired “mouthfeel and balance” or serving as a by-product for recovery [7,18,56,59]. The advantages of SCC include a high separation efficiency, a short residence time, minimal damage to the final product caused by temperature, good energy efficiency [7], the ability to work with highly viscous musts and wines, and the capability to completely dealcoholize a wine in a single pass. Non-volatile compounds remain unaffected compared to the original wine. However, this technique has disadvantages. It is necessary to heat the wine to approximately 38 °C, which will influence the quality of the final product [60].

Vacuum distillation is an adaptation of classical distillation that utilizes reduced pressure, enabling its application in removing ethanol from fermented beverages. This method aims to minimize the loss of heat-sensitive compounds. The vacuum created by a vacuum pump reduces the pressure inside the container and, consequently, the amount of heat required to reach the boiling point of the ethanol. This minimizes the damage caused by high temperatures, preserving heat-sensitive compounds to some extent [61,62]. Although this process reduces the production of smoky or burnt aromas in the final product, removing many esters and other volatile compounds along with the ethanol can be problematic since their boiling points are similar. Wines dealcoholized by vacuum distillation lack character compared to the original wine due to the loss of volatile aroma compounds [63]. Aguera et al. [64] demonstrated the feasibility of using vacuum distillation to remove ethanol from wine during fermentation, reducing wine quality when 2% (*v*/*v*) of the ethanol was removed. Vacuum distillation offers several advantages, including operating at low temperatures, concentrating the wine’s components, and eliminating the need for clarification. However, the main disadvantage is the need for special equipment and constant kinetics monitoring in case dealcoholization occurs during fermentation.

### 2.3. Enzymatic and Microbiological Methods

Enzymatic and microbiological methods are commonly employed to manage wines during various stages of vinification, including pre-fermentation, fermentation, and post-fermentation. The technologies used and the reduction in alcohol production differ in each phase (Table 3).

#### 2.3.1. Addition of Enzymes (Glucose Oxidase)

There are many enzymes used in the winemaking process: (i) in the pre-fermentative phase, for juice (pectolytic enzymes, cellulases, hemicellulases), for color (cellulases, hemicellulases, pectolytic enzymes), and aroma extraction (glycosidases), as well as for polyphenol (phenol oxidases) and sugar reduction (glucose oxidases); (ii) in the post-fermentative phase, the most important enzymes are used for clarification (proteases), for filtration (*β*-glucanases, cellulases, pectolytic enzymes), and for microbial stabilization (lysozyme); and in the aging phase, for mouthfeel extraction (*β*-glucanases and cellulases) [65].

Glucose oxidase (EC 1.1.3.4) is an enzyme mainly extracted from strains of *Aspergillus niger*. It can be used before the fermentation process as an effective technique to reduce the alcohol content of wines by lowering the glucose level in grape juice by up to 4%, thereby preventing ethanol formation during fermentation. The enzymatic and biochemical actions integrate two-step reactions. This enzyme initially converts *β*-D-glucose to D-glucono-lactone and hydrogen peroxide and then converts D-glucono-lactone into gluconic acid [66,67,68,69,70,71]. Several works have demonstrated the feasibility of this enzyme in reducing ethanol content, and these reductions seem to depend on the enzyme’s origin. A decrease of 0.7% (*v*/*v*) was found when using a glucose oxidase extracted from *Aspergillus oryzae* [69]; values of 2–4.3% (*v*/*v*) with one extracted from *Aspergillus niger* were rereported in the works of Rocker et al. [72] and Pickering [66], respectively. However, it must be highlighted that possible off-flavor development and modification of desirable organoleptic qualities, such as decreases in heptyl acetate concentration, certain alcohols with floral notes, and ketones with floral and fruity notes [73], may be essential limitations of its application in wines [65].

#### 2.3.2. Microbiological Methods

During fermentation, regarding the microbiological methods as a removal process of ethanol in wines, the following technologies are available: (i) use of non-*Saccharomyces cerevisiae* and *Saccharomyces* yeasts, with an alcohol content reduction of up to 2% *v*/*v*; (ii) use of modified genetically yeast strains, with an alcohol content reduction of up to 3.6% *v*/*v*; (iii) biomass reduction and arrested fermentation, with an alcohol content reduction of up to 4% *v*/*v*.

The utilization of non-*Saccharomyces cerevisiae* yeasts in sequential fermentation with Saccharomyces cerevisiae, in mixed fermentation, or single-strain fermentation for an ethanol reduction ranging from 0.2% to 2% (*v*/*v*) has been well documented in many works [74,75,76,77,78,79,80,81,82,83,84,85,86,87]. According to the information collected by Afonso et al. [28] in a recent review work, the most used non-*Saccharomyces cerevisiae* strains belong to *Candida* (*C*. *stellata*, *C*. *zemplinina*), *Hanseniaspora* (*H*. *osmophila*, *H*. *opuntiae*, *H*. *uvarum*), *Metschnikowia* (*M. pulcherrima*), *Pichia* (*P*. *kluyveri*, *P*. *kudriavzevii*), *Saccharomyces* (*S*. *uvarum*), *Schizosaccharomyces* (*S*. *pombe*), *Lachancea* (*L*. *thermotolerans*), and *Zygosaccharomyces* (*Z*. *bailii*, Z. *bisporus*, *Z*. *sapae*) species. Some non-*Saccharomyces cerevisiae* yeast strains can ferment less sugar or divert carbon metabolism to other pathways, avoiding excessive ethanol production during alcoholic fermentation [88].

Controlled aeration during fermentation can influence ethanol production. Non-*Saccharomyces* yeasts, such as *Metschnikowia pulcherrima* and *Torulaspora delbrueckii*, have demonstrated reduced ethanol yields under limited aeration conditions, with reductions of up to 2.0% (*v*/*v*) compared to standard anaerobic conditions [89,90]. However, the impact on wine quality varies depending on the yeast strain and aeration level [90,91].

Several specific non-*Saccharomyces* yeast strains are commonly used to lower the wine alcohol content: *M. pulcherrima, T. delbrueckii*, and *Zygosaccharomyces bailii*. These strains have been successfully used in sequential fermentations to lower ethanol levels while maintaining or enhancing wine quality [80,89,92]. These strains are selected based on their ability to grow and respire under the challenging conditions of grape-must [80].

The use of non-*Saccharomyces* yeasts can also affect a wine’s sensory profile. Sequential fermentations have been associated with increased concentrations of desirable compounds such as glycerol, esters, and higher alcohols, which can enhance the wine’s complexity and aroma [82,93,94,95]. However, some strains may produce excessive volatile acids or ethyl acetate, negatively affecting wine quality if not carefully managed [96].

The booming industrial application of non-*Saccharomyces* yeasts for reducing alcohol content requires a deeper understanding of their metabolic pathways and interactions with *S. cerevisiae*. Optimizing fermentation conditions, such as aeration and inoculation timing, is crucial to achieving consistent results without compromising wine quality [82,97].

Balancing the reduction in ethanol with preserving wine’s sensory and chemical properties remains a challenge. Future research should identify yeast strains that consistently produce wines with a reduced alcohol content while maintaining desirable sensory attributes [90,91]. Thus, non-*Saccharomyces* yeasts offer a viable approach to reducing the alcohol content in wine, with the potential to enhance sensory complexity. However, carefully selecting yeast strains and optimizing fermentation conditions are essential to maximize the benefits and minimize the drawbacks.

Ethanol respiration in yeasts, particularly in *Saccharomyces cerevisiae*, is a crucial process in both natural environments and industrial applications, such as wine dealcoholization. Understanding the mechanisms and factors influencing ethanol respiration is essential for optimizing industrial fermentation processes and enhancing yeast tolerance to ethanol. *S. cerevisiae* exhibits an overflow metabolism, where glucose is rapidly converted to ethanol even in the presence of oxygen—a phenomenon known as the Crabtree effect. This effect results from evolutionary adaptations that allow yeasts to outcompete other microbes by rapidly consuming glucose and producing ethanol, inhibiting competitors’ growth [98].

Yeasts have developed various adaptations to tolerate high concentrations of ethanol. These include changes at the genomic and proteomic levels, which affect energy metabolism pathways. Ethanol-adapted strains often switch from respiration to glycolysis and ethanol fermentation, highlighting the flexibility of yeast metabolism under stress [99].

Unusually, some *S. cerevisiae* can use ethanol as a carbon and energy source. *S. cerevisiae* strains S26 reduced acetic acid and ethanol in acidic wines. Vilela-Moura et al. [100] obtained a 37.5% reduction in ethanol under aerobic conditions and 13.5% under limited aerobic conditions. The same authors observed lower decreases in ethanol content (6–11.2%) using the strain immobilized in double-layer alginate-chitosan beads [101]. Understanding ethanol degradation pathways and the metabolic versatility of yeasts can facilitate modifications to wine composition in the post-fermentation stage. This advancement may lead to the development of new strategies to mitigate the adverse effects of climate change on viticulture [28].

Researchers have developed genetically modified yeast strains, such as those with partial deletions in the PDC2 gene, which can reduce ethanol production by up to 15% in certain strains without significantly affecting fermentation performance or wine quality [102]. Other strategies involve the non-expression of the alcohol dehydrogenase ADH enzyme, the overexpression of glycerol-3-phosphate dehydrogenase, and the expression, in yeasts, of non-yeast genes [28]. These modifications allow for the production of wines with regional characteristics and lower alcohol content. Genetic engineering has been used to redirect yeast metabolism away from ethanol production, although public acceptance of GMOs in food products remains a challenge [28,88]. However, any approach to reduce ethanol must ensure that the wine’s sensory properties and overall quality are not adversely affected [103].

Reducing yeast biomass is another approach to producing low-alcohol or non-alcoholic wine. This technique involves the periodic removal of yeasts, thereby decreasing the yeast population during must fermentation, which results in lower fermentation rates and prevents excessive ethanol formation [49,104,105,106,107]. Nevertheless, considerable amounts of unfermented sugars that remain in must represent a potential risk of spoilage and the formation of unpleasant flavors in the final product [106]. In some circumstances, when desired, this approach may produce sweeter and more pleasant alcoholic beverages [104].

The yeast fermentation activity can be rapidly arrested by cooling to 0 °C or pasteurization, e.g., temperature inactivation, and/or by removing yeast from the fermenting wort through filtration or centrifugation. This approach, like the previous one, can compromise the aromatic profiles of sweet wines with low alcohol or non-alcoholic content, as most aromatic compounds, such as monoterpenes and ethyl esters, are produced in large quantities by yeasts during extended fermentation times [108].

## 3. The Effect of Dealcoholization on the Sensory Profile of Wine

Reducing or eliminating alcohol content has been discussed more frequently in the wine sector lately for health reasons and due to the growing demand for options with less alcohol content [109]. However, this procedure raises questions regarding the impact on the sensory quality of the wine and, subsequently, on its acceptance by consumers.

**Figure 3:**
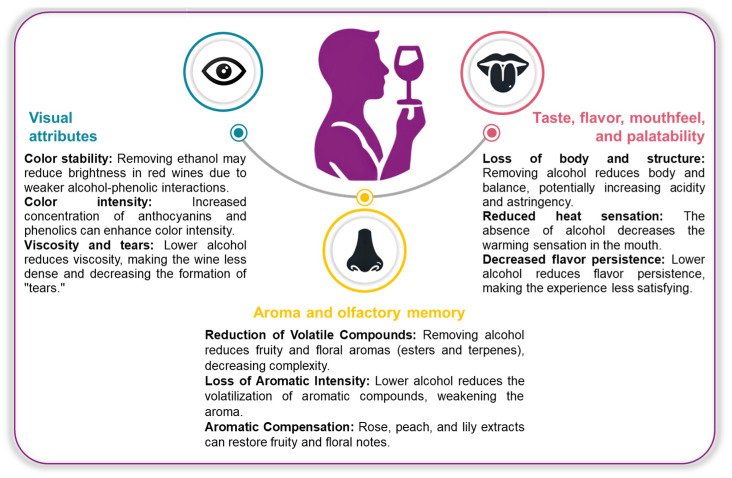
Sensory dimensions influenced by alcohol reduction in wine.

The presence of ethanol is crucial to the composition and sensory perception of wine. It influences sweetness and the volatilization of aromatic compounds. The partial reduction or complete removal of ethanol can alter several characteristics of this drink, influencing its appearance and aromatic taste profile (Figure 3).

Specific techniques are needed to avoid these changes [110]. Recent research suggests that reducing ethanol levels can significantly decrease volatile substances, such as esters and terpenes, responsible for wine’s floral and fruity aromas. This will hurt the aromatic profile and complexity of the final product [111]. It is also observed that the sensation of alcoholic heat and the viscosity of the wine will decrease in the absence of ethanol; this will result in enhancements of characteristics such as acidity and astringency that would be masked by alcohol [111].

### 3.1. Visual Attributes

Generally, dealcoholized wines tend to remain relatively unchanged. In some cases, brightness or color intensity may be lost, particularly in red wines. Interactions between alcohol and phenolic compounds will contribute to color stability, and the lack of ethanol may affect chromatic vividness [112]. Studies have shown that this process can intensify the color due to the concentration of anthocyanins and other phenolic compounds [111]. In their 2021 study, Sam et al. [18] discovered a fascinating phenomenon in Merlot (a red wine variety) where nearly complete dealcoholization—achieving a remarkable 94.9% reduction in ethanol content—resulted in a notable increase in color intensity.

### 3.2. Aroma and Olfactory Memory

Removing ethanol from wine can lead to noticeable sensory changes, primarily due to a considerable reduction in volatile compounds, predominantly esters, higher alcohols, acids, total terpenes, and C13-norisoprenoids. Ethanol also significantly shapes sensory characteristics [111,113,114]. Additionally, the process of ethanol removal may promote the binding of aroma compounds to proteinaceous substances, which can decrease their volatility and profoundly affect the sensory attributes of the final wine product [115]. Table 4 summarizes the most recent findings regarding the sensory changes in red or rosé wines following the alcohol reduction, namely regarding aroma perception.

The wine that has undergone dealcoholization through the various methods outlined in Table 4, particularly when the ethanol content reduction reaches up to 78% of the initial concentration, has exhibited a notable decline in its overall sensory profile. Specifically, the fruity and floral notes and the characteristics associated with red fruit and spices diminished significantly. This finding is supported by several studies, including those conducted by Sam et al. [18], Sun et al. [47], Ma et al. [49,116], Lisanti et al. [117], Corona et al. [118], and Puglisi et al. [123].

The reduction in these sensory parameters is primarily attributed to the substantial loss of volatile compounds during dealcoholization. For the majority of the wines analyzed, the most pronounced drop in olfactory qualities was observed in descriptors labeled “fruity/floral” and “red fruits”, both of which are particularly essential to the overall sensory quality of red and rosé wines (Table 4).

In a more nuanced observation of wine production techniques, partial dealcoholization—specifically removing 2% to 4% *v*/*v* ethanol—from Verdicchio red wine was achieved using osmotic distillation (OD). This process resulted in a noticeable decline in the wine’s characteristic honey attributes, as reported by Fedrizzi et al. [114]. This change suggests that even minor reductions in alcohol content can significantly affect the aromatic profile of specific wines.

In contrast, studies on Cabernet Sauvignon red wines that underwent partial dealcoholization demonstrated a different outcome. The percentage of alcohol content reduction ranged from 9.0% to 14.7% of the total amount through a combination method of reverse osmosis and ethanol permeation (RO-EP). Notably, the sensory profile changes observed in these wines were statistically non-significant, indicating that this method may preserve the essential characteristics of the wine despite the reduction in alcohol content, as outlined by Pham et al. [119,120].

Exploring the effects of nearly complete dealcoholization, Shiraz Sangiovese and Petit Verdot Sangiovese red wines underwent a remarkable reduction, with 98% of the total ethanol removed utilizing spinning cone column (SCC) technology. This extreme dealcoholization resulted in a discernible alteration in sensory attributes, notably a marked reduction in fruit aroma, flavor, and perceived hotness. Conversely, an increase in smoky and oxidized aromas was observed, suggesting that the method leads to significant changes in the overall wine profile, as detailed by Puglisi et al. [123]. This indicates that higher levels of dealcoholization can profoundly impact the wine’s sensory experience, raising questions about balance and quality in the final product.

Regarding white wine (not shown in Table 4), Ju et al. [124] found that Muscat wine distilled to a final concentration of ethanol of 6.15% *v*/*v* had less body, aroma intensity, and aroma purity compared to Muscat wine produced through controlled fermentation during alcoholic fermentation.

As can be seen from the examples in Table 4, the aromatic profile of wine is one of the aspects most significantly affected by dealcoholization. Alcohol acts as a vehicle for aromatic compounds, intensifying their volatilization and perception [125]. Removing alcohol can attenuate certain aromas, especially those associated with esters and terpenes, which evoke scents reminiscent of fruit and flowers. Additionally, it can increase the presence of undesirable compounds, such as herbaceous or acidic notes, rendering the wine sensorially unbalanced. Depending on the dealcoholization method, complexity and aromatic persistence may also be lost. Techniques such as vacuum distillation or reverse osmosis try to minimize these losses but do not permanently preserve the aromatic identity of the wine [111].

Reverse osmosis (RO) is widely used but is not entirely specific; it may exhibit variations in aroma and flavor during execution, which is more pronounced in red wines, where the complexity of volatile and phenolic compounds is greater [126]. Muñoz-González et al. [127,128] have shown that phenolics can alter the perception of aroma in red wines; when compared to the original wines, reduced-alcohol wines usually have poor sensory qualities, including a lack of wine body, flavor imbalance, diminished heat perception, bitterness, increased astringency, and excessive acidity.

According to Meillon et al. [129], the application of reverse osmosis in Merlot and Syrah red wine reduces the wine’s length in the mouth, enhancing perceptions of red fruits, as well as woody and blackcurrant notes (as measured by TDS), which is attributed to the reduced alcohol content. In a subsequent study, using the Syrah grape-variety wine, the authors [113] observed a decrease in heat and sweetness intensity (attributed to alcohol reduction), as well as a decrease in red fruit intensity and a decrease in persistence, complexity, and the number of aromas, accompanied by an increase in balance, harmony, and familiarity. Interestingly, they observed a decline in wine familiarity and harmony after a 4% (*v*/*v*) reduction.

On the other hand, vacuum distillation is a highly effective method for removing ethanol; however, it exhibits more pronounced sensory losses compared to reverse osmosis. This technique can cause a significant loss of esters and terpenes [111]. Additionally, pH and total acidity changes can alter taste perception, making the wine less balanced [111]. However, this technique has the advantage of preserving compounds such as polyphenols and anthocyanins, which are essential for the structure and color of wine [112]. The choice between these two techniques, or any other technique, will depend on the sensory profile of the wine we want to present.

Another relevant point is the consumer’s olfactory perception. The olfactory memory, especially those related to the appreciation of wine, will be permanently different since the lack of alcohol alters the interpretation of aromas and, therefore, the familiarity with a particular viticultural style [63]. On the other hand, wine tasters who would be prepared to identify characteristic aromas by the presence of ethanol will have to be educated to have an olfactory memory that can identify aromas in alcoholized wines.

Many studies have suggested that extracts, such as edible flower extracts (rose, peach, and lily), which have proven essential contributors to aromatic addiction, were added to control losses [49]. In the study by Ma et al. [49] and Sam et al. [130], the authors used extracts of rose (*Rosa chinensis*), peach (*Prunus persica*), and lily (*Lilium bulbiferum*) obtained through the maceration of petals in hot water (96 °C) and added them to the wine. Following the addiction to these extracts, the chemical analysis of the wines revealed a significant increase in volatile compounds, including esters (ethyl ethanoate and isoamyl octanoate) and terpene alcohols (linalool and geraniol). The sensory evaluation revealed greater aromatic intensity and fruity and floral notes in reconstituted wines. Moreover, rose (*Rosa chinensis*) was the best option for improving the aroma of dealcoholized Merlot red wine [49,130].

### 3.3. Taste, Flavor, Mouthfeel, and Palatability

Alcohol plays a significant role in the body’s feeling and the wine’s volume. With its partial or total removal, the perception can become more diluted and less structured; additionally, the normal balance between acidity, sweetness, and tannins can be disrupted, resulting in a wine that is either more astringent or more acidic [110,116], or both [17,117] (Table 4). The sensation of heat, typically associated with alcohol, dissipates, altering the product’s overall experience when tasted. Reducing alcohol can also affect the persistence of flavors, providing the consumer with a less pleasant experience, as there is no habituation when drinking this type of wine. Additionally, pH and total acidity changes can alter taste perception, making the wine less balanced [111,112]. Interestingly, acidity and astringency are not always interconnected. Longo et al. [122] verified in Shiraz red (7 months after bottling), both at middle harvest and late harvest, an increase in acidity and a decrease in astringency in the dealcoholized wines (Table 4).

These changes can be minimized using auxiliary methods employed by some companies, such as structuring agents or controlling phenolic ripening of the grapes before winemaking [121]. Among some structuring agents are polysaccharides such as Arabic gum and *β*-glucans, which aim to reduce the perception of astringency and acidity. To enhance the floral and fruity aroma, phenolic-free glycosides can be utilized, thereby improving the flavor without altering the astringency and acidity [49]. Other agents, such as mushroom extracts of the genus Ganoderma, can also enhance taste persistency [49]. These methods restore the body’s natural balance and provide a more balanced taste experience [116].

Notably, dealcoholization may also enhance the perception of the wine’s natural freshness and acidity, particularly in warmer regions where high sugar levels in grapes lead to a high alcohol content. A lower alcohol level will make the wine more refreshing and lighter, which improves its palatability, making it more versatile in gastronomic pairings [17,131,132], a point that will be discussed further.

## 4. Consumer Acceptance and Willingness to Pay

Consumer acceptance of dealcoholized wine is still evolving and depends on several factors, including consumption habits, sensory expectations, and price. Studies show that many consumers still think that the presence of alcohol is a fundamental parameter in the quality and complexity of the drink [133]. Consumers perceive low-alcohol wines positively, with ratings similar to those of standard wines in blind tastings. There is a growing market potential for low-alcohol wines, driven by health-conscious consumers and those seeking to reduce alcohol intake without compromising the wine-drinking experience [3,125]. However, there is a notable willingness to pay more for standard wines than for low-alcohol options, indicating a perception of higher value in traditional wines despite wine production involving advanced technologies and high-value investments [126,134].

Taste remains a crucial factor in the acceptance of low-alcohol wines. Consumers are more likely to accept these products if the taste is comparable to regular wines. Marketing efforts should focus on positioning low-alcohol wines as high-quality products rather than diet alternatives [134,135].

Interestingly, women and individuals who consume wine with meals are more inclined to purchase low-alcohol wines. Millennials and Baby Boomers, particularly those with mid-to-low incomes, also show interest in these products [136,137]. The ability to drink more without experiencing the adverse effects of a higher alcohol content is also a significant factor [7,136]. Situations such as needing to drive after drinking or preferring a lighter beverage option contribute to the preference for low-alcohol wines [135,136].

Marketing and consumer education will play a crucial role in promoting the value of this product. Promoting the benefits of low alcohol levels, such as enabling widespread consumption without essential side effects and the flexibility to live different lifestyles, can increase its market acceptance [138]. Less-experienced consumers have more difficulty understanding the differences between traditional and dealcoholized wines [132]. Effective marketing strategies are crucial for the success of low-alcohol wines. Emphasizing the health benefits, quality, and taste can help to capture a larger market share. The role of branding in influencing consumer preferences and willingness to pay is significant [134].

## 5. Health Benefits of Low- (Or No)-Alcohol Wines

The demand for non-alcoholic wine has been increasing significantly, driven by a growing emphasis on health and a response to the rising alcohol content in wine linked to climate change [28,139]. This trend is further reinforced by health-related restrictions on alcohol consumption, such as those associated with pregnancy, cardiovascular and hepatic disorders, and athletic performance [8,111]. Ethical and religious considerations also significantly determine consumer preferences [140]. In several countries, alcoholic beverages are subject to high import taxes, which vary according to their alcohol content [28,141,142].

All these combined factors increase interest in reducing the alcohol content in wines. According to the World Health Organization (WHO), alcohol is a toxic and psychoactive substance that induces addiction and poses significant health risks, being associated with 31 pathological conditions [143]. Alcohol consumption is responsible for 2.6 million deaths annually worldwide. It contributes to various health issues, including cardiovascular problems such as cardiomyopathy, coronary artery disease, hypertension, and stroke [111,125,144]. During its metabolism, alcohol is oxidized to acetaldehyde, a highly reactive compound mediating much of the ethanol-induced toxicity, potentially intensifying the development of chronic diseases, including cardiovascular disorders [145]. As a result, the consumption of low- and zero-alcohol wines has gained substantial popularity in recent years, with data indicating that 9% and 13% of consumers prefer these beverages, viewing low-alcohol wine as a healthier and safer alternative [28].

**Figure 4:**
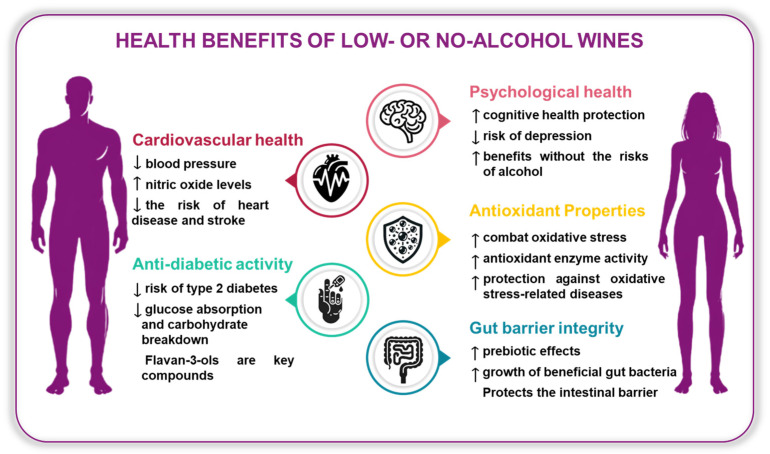
Health benefits of low- (or no)-alcohol wines.

The consumption of low- and zero-alcohol wines has been linked to various potential health benefits, including enhanced cardiovascular health, antioxidant effects, improved gut microbiota composition, and anti-diabetic properties, making them a promising choice for those seeking pleasure and well-being (Figure 4) [7].

### 5.1. Antioxidant Properties

Wine consumption has been linked to numerous health benefits, primarily due to its bioactive compounds, such as polyphenols, which possess antioxidant properties. Although alcohol is considered a risk factor for several diseases, studies suggest that wine polyphenols may play a protective role, particularly in modulating oxidative stress, which is linked to conditions such as heart disease, cancer, diabetes, and Alzheimer’s [146]. With the growing interest in low-alcohol alternatives, alcohol-free wine has gained attention as an option that preserves the antioxidant benefits while avoiding the adverse effects of alcohol [28].

A study conducted by Noguer and his team [147] was one of the first to demonstrate that the increase in antioxidant enzyme activity is not due to the alcohol content in wine but rather to its polyphenolic composition. This study involved healthy individuals who followed a low-phenolic diet to minimize the interference of other antioxidant compounds. During the study, half of the participants consumed 300 mL of dealcoholized wine daily for one week, while the other half followed the same diet but did not consume wine. After this period, the groups were switched to evaluate the effects of dealcoholized wine on antioxidant activity. The results showed that during the low-phenolic diet, there was a decrease in the activity of the enzymes glutathione reductase, superoxide dismutase, and catalase. However, after consuming alcohol-free wine, the activity of these enzymes significantly increased. Significant changes were observed on the third day of intervention for glutathione reductase and superoxide dismutase and the seventh day for catalase. The authors concluded that the beneficial effects observed were not attributed to alcohol itself but rather to the polyphenolic compounds present in alcohol-free wine, which may contribute to enhancing antioxidant activity in the body. This study suggests that consuming alcohol-free wine could be an excellent source of antioxidants to protect individuals suffering from oxidative stress (such as cancer, diabetes, Alzheimer’s, etc.) who should avoid or prefer not to consume alcohol. In an in vivo study, Mihailovic-Stanojevic et al. [148] reported an enhancement of antioxidant efficiency. They reduced the susceptibility of plasma to lipid peroxidation in spontaneously hypertensive rats with alcohol-free red wine. They also observed that the concentration of phenolic compounds, known for their beneficial effects on human health [146], did not suffer significant changes during the removal of alcohol from standard wine. This suggests that the phenolic compounds present in wine contribute to mitigating oxidative stress.

### 5.2. Cardiovascular Health

Coronary heart disease and stroke are significant causes of mortality and disability in developed countries, with atherosclerosis being the leading cause of coronary heart disease [146,149]. The relationship between wine consumption and cardiovascular health remains a topic of debate. While moderate alcohol intake has historically been associated with a lower cardiovascular risk, recent evidence suggests that the benefits of red wine are primarily attributed to its polyphenol content rather than its alcohol component. Chiva-Blanch et al. [150] demonstrated that in high-risk individuals, consuming dealcoholized red wine for four weeks significantly reduced systolic and diastolic blood pressure by 5.8 mmHg and 2.3 mmHg, respectively, in addition to increasing plasma nitric oxide levels. These findings suggest that alcohol may attenuate the blood pressure-lowering effects of red wine polyphenols. Given that even modest blood pressure reductions can substantially decrease the risk of coronary heart disease and stroke, dealcoholized red wine may represent a promising strategy for hypertension prevention. In a subsequent study, Chiva-Blanch et al. [151] assessed the effects of consuming red wine (30 g alcohol/day) and dealcoholized red wine for four weeks in men at high cardiovascular risk. The results indicated that dealcoholized red wine, primarily due to its polyphenols, significantly improved insulin resistance, similar to conventional red wine. Moreover, unlike alcoholic red wine, dealcoholized red wine did not alter lipoprotein levels but positively affected metabolic health. These findings highlight that polyphenols in dealcoholized wine can contribute to cardiovascular and metabolic health, even in the absence of alcohol. Blalock et al. [152] suggest that consuming low-alcohol wine or gradually reducing alcohol intake may decrease systolic blood pressure, highlighting the potential of dealcoholized or lower-alcohol wines as beneficial alternatives for cardiovascular health. Similarly, Lamont et al. [153] demonstrated that reducing the alcohol content of wine from 12% to 6% did not affect its antioxidant properties or cardioprotective effects against ischemic injury. The removal of alcohol also did not change these properties, suggesting that polyphenols, particularly resveratrol, are primarily responsible for the observed benefits. Preclinical studies indicate that both conventional red wine and alcohol-free wine can reduce atherosclerotic plaque formation, even in the context of hypercholesterolemia. This protective effect is associated with antiplatelet mechanisms, inhibition of endothelial cell adhesion, and stimulation of nitric oxide (NO) production [154].

In a study with well-controlled type 2 diabetes patients [155], it was observed that ingesting red wine (with women consuming 230 mL of red wine per day, approximately 24 g of alcohol, and men consuming 300 mL per day, around 31 g of alcohol) increased both systolic and diastolic blood pressure during the day, while dealcoholized red wine did not induce this adverse effect. Furthermore, dealcoholized red wine did not increase heart rate, as observed with the consumption of alcoholic red wine. An increase in heart rate is considered a cardiovascular risk factor. Additionally, dealcoholized red wine did not negatively affect glycemic control, whereas standard red wine neither improved nor worsened glycemic control in individuals with diabetes. These findings suggest that for individuals with type 2 diabetes, dealcoholized wine may be a safer and more effective alternative for cardiovascular health.

A more recent study [156] demonstrated that reducing alcohol consumption significantly decreased blood pressure, particularly in morning blood pressure levels. After six months of intervention, 55.6% of participants who reduced their alcohol intake achieved blood pressure levels below 135/85 mmHg, compared to only 16.7% in the control group. The average alcohol consumption was significantly lower in the intervention group (256 ± 206 g/week) compared to the control group (413 ± 260 g/week). These findings suggest that adopting lower-alcohol beverages, such as reduced-alcohol wine, maybe a practical approach to lowering hypertension and cardiovascular risks associated with excessive alcohol consumption.

While the impact of wine on cardiovascular health remains a topic of debate, there is a consensus that both low-alcohol and alcohol-free wines can offer cardiovascular benefits. However, further research is needed to elucidate the interactions between various wine components, such as polyphenols, antioxidants, and alcohol, to understand their effects on cardiovascular health fully.

### 5.3. Anti-Diabetic Activity

According to data from the World Health Organization, *diabetes mellitus* will affect approximately 500 million people by 2025. This complex metabolic condition is associated with a range of complications, including dysfunctions in the retina, kidneys, limbs, heart, nerves, and blood vessels [157], reducing the quality of life and ultimately leading to death. Several studies suggest that moderate consumption of wine is associated with a lower risk of developing type 2 diabetes [111,158].

The anti-diabetic properties of dealcoholized Portuguese red wine were studied in vitro assays [159]. The authors assessed the ability of this wine to inhibit two enzymes: α-glucosidase, which catalyzes the release of glucose from disaccharides, and α-amylase, which breaks down complex carbohydrates into smaller sugars. It was demonstrated that both dealcoholized red wine and red wine fractions obtained through solid-phase extraction exhibited potent inhibitory activity against these enzymes, indicating that the phenolic compounds present in those samples may reduce the absorption of sugars in the intestine, a beneficial effect for the prevention and management of diabetes. Additionally, the study identifies flavan-3-ols (monomeric and oligomeric) as the main compounds responsible for this activity.

### 5.4. Gut Barrier Integrity and Polyphenolic Interactions

Moderate consumption of red wine can directly impact the microbiota, with polyphenols promoting the growth of health-related species and exerting antimicrobial effects against pathogenic bacteria [146]. These beneficial effects have shown the potential of red wine polyphenols to improve gut health and modulate microbiota composition, with compounds such as flavonoids and resveratrol enhancing the growth of probiotic bacteria [160]. In recent years, studies have been conducted on the effects of alcohol-free wine on gut microbiota health. Queipo-Ortuño et al. [161] investigated the impact of dealcoholized red wine on gut microbial groups. In their in vivo study, ten healthy volunteers consumed 272 mL of alcohol-free red wine daily for 20 days. The results showed a significant increase in the number of beneficial microbial species, such as *Prevotella*, *Enterococcus, Bifidobacterium, Bacteroides, Bacteroides uniformis, Eggerthella lenta,* and *Blautia coccoides–Eubacterium rectale* groups, along with improvements in health markers, including reduced levels of triglycerides and cholesterol. This suggests that the polyphenols present in alcohol-free red wine can positively affect gut health, even without alcohol, which suggests possible prebiotic benefits. In another study conducted by Moreno-Indias et al. [162], the effects of regular consumption of red wine and dealcoholized red wine on patients with Metabolic Syndrome were investigated regarding the modulation of the gut microbiota. After one month of consumption of both wines, a significant improvement in the fecal microbiota composition was observed. The polyphenols from the dealcoholized wine exhibited a prebiotic effect, promoting the growth of beneficial bacteria, such as *Bifidobacterium* and *Lactobacillus*, while reducing the abundance of inflammation-associated bacteria, such as *Bacteroides*. Furthermore, the intake of both alcoholic and dealcoholized wine resulted in a significant reduction in blood pressure, cholesterol, and glucose levels. It promoted the production of short-chain fatty acids, such as butyrate, contributing to gut health. Further studies using a non-alcoholic red wine extract (RWE) rich in polyphenols, obtained from Portuguese wine from the Douro region, composed of the Touriga Nacional, Touriga Francesa, Touriga Franca, and Tinta Roriz varieties, demonstrated its protective effects against *Escherichia coli* 270-induced cytotoxicity in human intestinal HT-29 cells [163]. The extract was rich in catechins, oligomeric procyanidins, anthocyanins (mainly malvidin-3-glucoside), phenolic acids, and gallic and syringic acid. The RWE exhibited protective effects by inhibiting the activity of the exotoxin secreted by the *E. coli* strain, likely the cytotoxic necrotizing factor (CNF-1), which is responsible for cell death. Interestingly, the protection did not involve inhibition of bacterial adhesion, suggesting that the polyphenols may interact with the exotoxin or the epithelial cells to prevent its toxic effects. This study highlights the potential of non-alcoholic red wine polyphenols as a natural therapeutic approach for protecting the intestinal mucosa against bacterial toxins.

### 5.5. Impact on Psychological Health

The impact of non-alcoholic wines on psychological health is not directly addressed in any study. However, insights can be drawn from research on alcoholic wine consumption and its psychological effects, which may offer indirect implications for non-alcoholic variants.

Moderate wine consumption has been associated with a reduced risk of cognitive decline in older adults. A meta-analysis found that wine drinkers had a lower relative risk of cognitive decline than non-drinkers, suggesting potential protective effects [164].

Moderate wine consumption, precisely two to seven drinks per week, has been linked to a lower incidence of depression. This suggests that certain compounds in wine, possibly polyphenols, might contribute to mental well-being [165].

While the studies focus on alcoholic wine, the potential benefits may be partially attributed to non-alcoholic components, such as polyphenols and antioxidants, present in wine. Non-alcoholic wines, which retain these compounds, offer similar benefits without the risks associated with alcohol consumption. However, direct research on non-alcoholic wines is needed to confirm these effects.

## 6. Food Pairing, the Gourmet Experience, and Low- (Or No)-Alcohol Wines

Historically, low-alcohol wines were consumed daily in regions like Beaujolais and the Loire Valley, where Muscadet and Anjou’s rosés were popular. These wines were integral to traditional food and wine pairings, providing a balanced experience that complemented various dishes [166]. Moreover, adding elements like essential oils in novel low-alcohol wine products enhances aroma and taste, contributing to a richer gourmet experience [167].

Successful food and wine pairings often involve balancing sensory attributes, including flavor intensity, texture, and complexity. Studies have shown that appropriate pairings can enhance consumer liking and sensory complexity, usually correlating with higher expected prices and positive emotional responses [168,169]. Sensory complexity and balance are crucial, with a slight dominance of wine often preferred in pairings [168]. Table 5 presents some pairing experiences with world dishes and alcoholic wine.

Various factors influence successful food and wine pairings, including sensory attributes, consumer preferences, and cultural traditions. Successful pairings often involve a slight dominance of wine over food, which enhances liking and sensory complexity. This balance is crucial for creating an enjoyable experience (Table 5) [168]. For instance, pairings that increase the overall flavor intensity and complexity are generally preferred. The interaction between food and wine can change the taste attributes, leading to a more enjoyable experience [168,169]. Also, a wine’s sweetness, acidity, and tannin levels significantly impact the perception of match with food. These characteristics must be considered to achieve a harmonious pairing [174].

The food and wine pairing field is evolving, with new approaches such as computational gastronomy and heterogeneous graphs like WineGraph to predict successful pairings based on taste descriptors [175,176]. These innovations aim to refine pairing strategies and enhance consumer experiences, though challenges remain in generalizing pairing principles across different beverages and cultural contexts [177].

Many successful pairings are based on cultural and geographical matches, internalized as semantic knowledge among consumers. These pairings often emerge naturally and are widely accepted [175]. Pairing regional wines (Table 5) with local foods can promote sustainability and enhance the gastronomic experience, contributing to the local economy and tourism [170]. Expert knowledge and training in food and wine pairing can significantly impact the perceived quality of pairings. Experts often use a hierarchical approach to match components, textures, and flavors [178,179].

In addition to wine and food pairing, wine has three key uses in the kitchen: a marinade constituent, a cooking liquid, and a flavoring in a final dish. Wine’s function in cooking is to intensify, enhance, and accent the flavor and aroma of food. As with any seasoning used in cooking, care should be taken in the quantity of wine applied; too little is trivial, and too much will be overshadowed. Neither extreme is desirable. A small amount of wine will improve the dish’s flavor [180].

The components of wine, such as ethanol, polyphenols, and other matrix elements, significantly affect the release and perception of volatile aroma compounds. These components can alter the headspace concentration of volatiles, thereby influencing aroma perception [181,182,183]. Typically, ethanol, glucose, and glycerol are the primary components that influence volatile partitioning. Increased ethanol concentration tends to reduce the headspace concentration of volatile compounds, potentially suppressing specific aroma attributes [184]. Conversely, glucose can enhance the concentration of volatiles in the headspace [184]. This happens when we taste a wine in a proper tasting glass [185]. Polyphenols interact with volatile organic compounds, affecting their volatility and solubility and influencing aroma perception under orthonasal and retronasal conditions [186].

Alcohol modifies the sensory perception of wine’s aromatic attributes and the detection of volatile compounds. Humans experience the presence of alcohol in food through the senses of taste, smell, and touch. However, what will the main interactions be if we use low-alcohol wine for cooking? Although the wine does not contain alcohol, it contains chemical components that affect its texture and mouthfeel, such as tannins and acids. Tannins affect a wine’s texture and cause a drying sensation in the mouth, known as astringency. Astringency is primarily attributed to the interaction between tannins and salivary proteins, resulting in protein–tannin complexes that precipitate, reducing lubrication in the mouth [187]. This interaction is influenced by factors such as pH, alcohol content, and the presence of other polyphenols [188]. Table 6 presents three examples of non-alcoholic wine options and their suggested pairing dishes. The table offers a selection of low- or no-alcohol wines and their ideal pairing dishes, highlighting the sensory and flavor profiles that make these combinations appealing. The references indicate the sources of information on the wine types and their characteristics, ensuring that the pairings are well informed and based on current research.

When cooking with wine, the wine tannins become attracted to the proteins in meat rather than the proteins in our saliva, which makes the wine appear softer in the mouth [178,179]. According to Joachim and Schloss [180], when making a sauce with Cabernet Sauvignon, the tannins become more concentrated as the sauce reduces. The sauce may develop an astringent taste without enough protein and fat to balance the tannins. For a vegetarian sauce, a less tannic red wine, such as Pinot Noir, or a white wine may be a better choice.

Acidity is another essential chemical and sensory factor to consider when cooking with wine. For example, pairing a tomato sauce with a low-acid red wine like Merlot can make the wine seem flat, as the high acidity of the tomatoes overpowers it. This happens because Merlot typically has lower acidity and cannot withstand the tomatoes’ tartness. On the other hand, a classic Chianti, made from Sangiovese grapes, may not be ideal either—its naturally high acidity can amplify the tartness of the tomato sauce, making the dish overly sharp [178,179].

And what about aroma sensations? Is the wine alcohol content important for an integrative combination with the food aromas? Alcohol shares structural similarities with sugar molecules and has a mildly sweet taste. However, it can irritate at high concentrations, creating a pungent, “hot” sensation in the mouth and nose [190,191]. As a volatile compound, alcohol has a distinct aroma and interacts chemically with other volatile molecules. In high concentrations, it can trap these compounds in foods and beverages, reducing their release into the air. Conversely, at very low concentrations—around 0.5–1% (*v*/*v*) or less—alcohol enhances the release of fruity esters and other aromatic compounds, intensifying their scent [141].

According to Harrington [178,179], it is worth considering wines with similar taste characteristics when creating a dish with one or two dominant flavors. For example, Pinot Noir—particularly from Burgundy—is known for its earthy, mushroom-like aromas, making it a natural pairing for a dish featuring fresh, sautéed mushrooms. A bright dish with a splash of lemon juice may complement a wine with crisp citrus notes, such as Sauvignon Blanc. Similarly, a creamy shrimp dish will likely pair well with a rich, buttery Chardonnay [178,179]. However, some experts argue that the aroma of wine is less critical when selecting one for cooking. They suggest that non-volatile compounds in red wine play a crucial role in meat stock preparation and that the cooking method significantly influences the flavor development of a red wine reduction [192]. Other examples are presented in Table 6.

## 7. Final Remarks

Low- and no-alcohol wines are characterized by their reduced alcohol content and are classified variably across different regions. The increasing trend toward low-alcohol and non-alcoholic beverages is primarily driven by health-conscious consumers seeking quality and variety. This shift is observable across multiple markets, reflecting significant changes in beverage consumption patterns that align with healthier lifestyle choices. The demand for these beverages is expanding, with craft and premium options gaining noticeable popularity.

Low- and no-alcohol wines offer a promising alternative for individuals looking to reduce their alcohol intake while maintaining social interactions. These products align with overarching health-conscious trends and present potential economic benefits for producers. Nevertheless, the willingness of consumers to pay premium prices for these options poses a challenge, and further research is essential to comprehend the long-term social and health implications fully.

Various legal definitions and consumer perceptions significantly influence the production and regulation of low-alcohol beverages. Global standardization in definitions and labeling is urgently needed to ensure consumer safety and enhance market clarity. Additionally, improved analytical methods and regulatory oversight are necessary to uphold compliance and protect consumer interests.

The application of genetically modified organism (GMO) yeast strains presents a viable approach to reducing the alcohol content in wine, leading to substantial decreases in ethanol production. However, challenges related to consumer acceptance and regulatory constraints persist. Non-GMO alternatives, including different yeast strains and innovative fermentation techniques, may offer promising solutions that consumers will likely embrace. Both strategies aim to respond to the demand for lower-alcohol wines without sacrificing quality.

Furthermore, due to their impact on the sensory profile of wine, dealcoholization processes—whether viticultural, physical, enzymatic, or microbiological—should be integrated with complementary methods to minimize any undesirable alterations. Consumer acceptance will likely hinge on the sensory quality of these products and their evolutionary trajectory. As technological advancements occur, there is a need for the development of more effective communication strategies. The wine industry should continue to innovate and present more appealing options that cater to public preferences.

While low- and no-alcohol wines represent a significant market opportunity, their success will depend on addressing consumer perceptions regarding quality and taste, understanding demographic preferences, and implementing strategic marketing initiatives to enhance product acceptance and foster market growth. Additionally, incorporating low- and no-alcohol wines into gourmet food pairing experiences offers a nod to traditional practices while resonating with contemporary movements toward sustainability and health. The sensory attributes, contextual considerations, and innovative pairing approaches will continue to influence the evolution of this culinary art.

## Figures and Tables

**Table 1:** Viticultural techniques for alcohol reduction: fundamentals, benefits, and limitations.

Wine Dealcoholization Techniques		Fundamentals	Advantages	Disadvantages	References
Viticultural practices	Early harvesting of grapes	Harvest grapes earlier than usual or mix early- and fully ripened grapes to reduce sugar content and thus lower the alcohol content.	Reduces alcohol by up to 3% *v*/*v*; maintains acceptable acidity and sensory properties in some varieties.	It may cause unripe flavors, higher acidity, and incomplete development of aroma precursors.	[13,14,15,16,17]
	Reduction in leaf area	Limits sugar accumulation in grapes by reducing photosynthetic activity through strategic leaf removal or pruning.	Reduces alcohol content while preserving flavor and phenolic compounds; enhances certain aroma compounds in some varieties.	Effectiveness is variety-dependent and may lead to yield reductions or altered aromatic balance if not appropriately managed.	[18,19,20,21,22,23,24,25,26,27,28,29,30,31,32,33,34]
	Late pruning	Delays winter pruning to postpone ripening, reducing sugar accumulation and enhancing phenolic maturity.	It improves acidity and phenolic content, lowers alcohol content, and enhances the sensory profile.	Reduces yield; variable effects depending on grape variety; may impact economic viability.	[19,20,35,36,37,38,39,40,41]

**Table 2:** Physical techniques for alcohol reduction: fundamentals, benefits, and limitations.

Wine Dealcoholization Techniques		Fundamentals	Advantages	Disadvantages	References
Physical Methods	Membrane processes	Use of selective membranes to separate ethanol from wine via physical processes such as pervaporation (PV), osmotic distillation (OD), nanofiltration, or reverse osmosis (RO).	It can be applied at low temperatures, is energy-efficient, and is scalable; some methods also preserve wine quality.	Aroma losses, especially with PV and OD, may require adding water (RO), which has varying impacts on sensory quality.	[18,42,43,44,45,46,47,48,49,50,51,52,53,54,55]
	Thermal processes	Heat can be used under a vacuum or via spinning cone columns to remove volatile compounds, including ethanol.	High efficiency, low-temperature damage, and potential for complete dealcoholization, making it applicable to viscous wines.	Removes desirable aromas, resulting in quality loss, and requires specialized equipment and monitoring.	[7,18,56,57,58,59,60,61,62,63,64]

**Table 3:** Enzymatic and microbiological techniques for alcohol reduction: fundamentals, benefits, and limitations.

Wine Dealcoholization Techniques		Fundamentals	Advantages	Disadvantages	References
Enzymatic Methods (Glucose Oxidase)		Enzymatic oxidation of glucose before fermentation to limit ethanol formation using glucose oxidase.	Effective ethanol reduction (up to 4.3% *v*/*v*); pre-fermentation use; controlled enzymatic action.	Possible off-flavor development; reduction in desirable aromatic compounds.	[65,66,67,68,69,70,71,72,73]
Microbiological Methods	Non-*Saccharomyces* yeasts	Use non-*Saccharomyces* yeasts in pure, mixed, or sequential fermentations to reduce ethanol through altered metabolic pathways.	Reduction of up to 2% *v*/*v* ethanol; enhanced sensory complexity; increased aroma diversity.	Risk of volatile acidity or ethyl acetate formation; strain-dependent performance; strict fermentation management needs.	[28,74,75,76,77,78,79,80,81,82,83,84,85,86,87,88,89,90,91,92,93,94,95,96,97]
	*S. cerevisiae* ethanol respiration	Use ethanol respiration and metabolic versatility in *S. cerevisiae* to consume ethanol under aerobic or limited-aerobic conditions.	Ethanol reductions of up to 37.5% under aerobic conditions; improved acetic acid levels; potential post-fermentation application.	Strain-specific; requires oxygenation; immobilized forms less effective; practical application still experimental.	[28,98,99,100,101]
	Genetically modified yeasts	Use of GMO yeast strains modified to downregulate ethanol production pathways or overexpress alternative metabolite pathways.	Reductions of up to 15% ethanol maintained fermentation performance and regional characteristics in some strains.	Consumer acceptance of GMOs is limited. Preserving wine sensory quality is essential, and regulatory issues must be addressed.	[28,88,102,103]
	Biomass reduction and arrested fermentation	Periodic removal of yeasts or sudden fermentation arrest (cooling, filtration) to limit ethanol production.	Can produce sweet wines with lower alcohol; simple physical intervention; avoids complete fermentation.	Risk of spoilage due to residual sugars; loss of key aroma compounds; quality may suffer if poorly controlled.	[49,104,105,106,107,108]

**Table 4:** Overview of aroma and mouthfeel attributes of red and rosé wines with different methods of dealcoholization. RO-EP/MC, reverse osmosis–evaporative perstraction/membrane contactor; SCC, spinning cone column; RO, reverse osmosis; VD, vacuum distillation; OD, osmotic distillation; PV, pervaporation.

Dealcoholization Process	Wine Type and Percentage of Alcohol Reduction	Findings on Olfactory Characteristics	Ref.
RO and VD	Merlot (red)—94.9% Pinot Noir (rosé)—94.3%	The perception of red fruit notes, aroma intensity, and overall acceptability decreased. Wine body, hotness, and bitterness also decreased. Color intensity, astringency, and acidity perception increased.	[18]
RO	Pinot Noir (rosé)—94.3%	The perception of red fruit notes, aroma intensity, and overall acceptability decreased. Decrease in acidity, wine body, and astringency perception (mouthfeel). There was no effect on color intensity and sweetness.	[49,116]
OD	Aglianico (red) 32.3% (A)—36.1% (B)	Cherry and red fruit notes decreased (A). Decrease in cherry, red fruits, and flower notes (B). Astringency and acidity increased (A and B).	[117]
	Montepulciano d’Abruzzo (red)—78.8%	Spicy notes and red fruit notes decreased. There is no significant effect on color intensity. Overall acceptability and acidity increased.	[118]
	Verdicchio (red)—2.0 to 4.0%	The honey scent was depleted. There is no significant effect on acidity, saltiness, and bitterness. Wine body and persistence decreased.	[114]
RO-EP/OD/MC	Cabernet Sauvignon (red)—14.7 to 9.0%	There was no effect on the overall intensity. There was a slight decrease in the flavor of dried fruit and chocolate.	[119,120]
	Shiraz red (7 months after bottling) Middle harvest—22.2% Late harvest—18.2 to 36.4%	No significant differences were found in eucalyptus, red fruit, nutty/almonds, green olive, floral, and plum aromas. There was a decrease in dark fruit, raisin/prune, black pepper, and herbaceous aromas, with overall aroma density. Increase in acidity and decrease astringency.	[121]
	Verdelho red (late harvest)—48.1%	No significant differences in buttery/nutty, citrus, apple/pear aroma, and acidity Decrease in tropical fruit, hay, peach/apricot, herbaceous, floral, overall aroma density, bitterness taste	[122]
	Petit Verdot—19.2%	There are no significant differences in eucalyptus, green paper, floral, dark fruit, cooked aroma, and acidity. The presence of red fruit, raisins, and prunes decreased, as did the overall aroma intensity.	
PV	Cabernet Sauvignon (red)—96.0%	High retention of fruit aroma, producing wine with better smell and taste.	[47]
SCC	Shiraz Sangiovese (red)—98.0% Petit Verdot Sangiovese—97.9%	There was a decrease in fruit aroma, flavor, and hotness but an increase in smoke and oxidized aromas. There were no significant changes in the woody aftertaste.	[123]

**Table 5:** Pairing experiences with wine type, pairing dish, and some key insights.

Wine Type	Pairing Dish	Key Insights
Regional Wines	Regional Dishes (Algarve, Portugal)	Experts selected regional wines to pair with regional dishes, promoting sustainability and quality in tourism [170].
Australian Shiraz	Complex Food Samples	Pairings with Shiraz increased flavor intensity and were preferred by consumers for their sensory complexity [168].
Shade-Grown White Wine	Sashimi (Japanese Cuisine)	Shade-grown grapes enhanced the palatability of sashimi, similar to Japanese sake [171].
Riesling	Chinese Cuisines	Riesling was preferred for pairing with various Chinese dishes, showing significant interaction between wine and cuisine [172].
Tannic Red Wine	Spicy Korean Dishes	Tannic red wines pair well with spicy Korean dishes despite the challenges posed by spiciness and intense flavors [173].

**Table 6:** Examples of non-alcoholic or low-alcohol wine options and suggested pairing dishes and notes.

Wines	Low or No	Suggested Pairing Dish	Notes
Muscadet (Loire)	No-alcohol wines	Seafood dishes, oysters or shellfish	Muscadet is known for its crisp and refreshing profile, making it an excellent match for seafood [166].
Rosé of Anjou		Light salads or grilled vegetables	The light and fruity nature of Rosé of Anjou complements fresh and light dishes [166].
Piquette		Rustic, hearty meals	Piquette, a low-alcohol beverage, pairs well with simple, hearty meals, reflecting its origins as a drink for field workers [166].
Chardonnay ^1^	Low- alcohol wines	Creamy pasta dishes	Chardonnay, with reduced alcohol content, maintains a balance that complements creamy textures [84,189].
Shiraz ^1^		Grilled meats or barbecue	The robust flavors of Shiraz, even with reduced alcohol, pair well with hearty, grilled dishes [84,189].
Merlot ^1^		Roasted chicken or turkey	The red fruit flavors in reduced-alcohol Merlot enhance the taste of roasted poultry [189].
Low-Alcohol Sangria-Type Wine ^2^		Tapas or spicy dishes	The aromatic profile of sangria-type wines with essential oils pairs well with flavorful, spicy foods [167].

^1^ With a 1–2% (*v*/*v*) content lower than in the wines obtained with *S. cerevisiae* strains. ^2^ With 6% ethanol (*v*/*v*).

## Data Availability

This study did not create or analyze new data, and data sharing is not applicable to this article.

## References

[1] Anderson K. (2023). The emergence of lower-alcohol beverages: The case of beer. J. Wine Econ..

[2] Myles C., Weil B., Wiley D., Watson B. (2022). Representations of Low(er) Alcohol (Craft) Beer in the United States. Nutrients.

[3] Bucher T., Deroover K., Stocley C. (2018). Low-alcohol wine: A narrative review on consumer perception and behaviour. Beverages.

[4] Schienkiewitz A., Haftenberger M., Mensink G. (2020). Time trends of non-alcoholic beverage consumption among adults in Germany, 1990–2011. Nutr. J..

[5] Mesirow M., Welsh J. (2015). Changing beverage consumption patterns have resulted in fewer liquid calories in the diets of US children: National Health and Nutrition Examination Survey 2001–2010. J. Acad. Nutr. Diet..

[6] Jones A., Kirkpatrick S., Hammond D. (2019). Beverage consumption and energy intake among Canadians: Analyses of 2004 and 2015 national dietary intake data. Nutr. J..

[7] Silva P. (2024). Low-Alcohol and Nonalcoholic Wines: From Production to Cardiovascular Health, Along with Their Economic Effects. Beverages.

[8] Okaru A., Lachenmeier D. (2022). Defining No and Low (NoLo) Alcohol Products. Nutrients.

[9] OIV (2025) 2 Months, 12 Resolutions: Dealcoholisation of Wines. OIV-OENO 394A-2012. https://www.oiv.int/press/12-months-12-resolutions-dealcoholisation-wines.

[10] Saliba A., Ovington L., Moran C. (2013). Consumer demand for low-alcohol wine in an Australian sample. Int. J. Wine Res..

[11] Anderson P., Kokole D., Llopis E. (2021). Production, Consumption, and Potential Public Health Impact of Low- and No-Alcohol Products: Results of a Scoping Review. Nutrients.

[12] Vasiljevic M., Couturier D., Marteau T. (2017). Impact of low alcohol verbal descriptors on perceived strength: An experimental study. Br. J. Health Psychol..

[13] Bindon K., Varela C., Kennedy J., Holt H., Herderich M. (2013). Relationships between harvest time and wine composition in *Vitis vinifera* L. cv. Cabernet Sauvignon 1. Grape and wine chemistry. Food Chem..

[14] Asproudi A., Ferrandino A., Bonello F., Vaudano E., Pollon M., Petrozziello M. (2018). Key norisoprenoid compounds in wines from early-harvested grapes in view of climate change. Food Chem..

[15] de Toda F.M., Balda P. Decreasing the alcohol level in quality red wines by the “double harvest” technique. Proceedings of the 17th International Symposium Giesco.

[16] Piccardo D., Favre G., Pascual O., Canals J.M., Zamora F., González-Neves G. (2019). Influence of the use of unripe grapes to reduce ethanol content and pH on the color, polyphenol and polysaccharide composition of conventional and hot macerated Pinot Noir and Tannat wines. Eur. Food Res. Technol..

[17] Kontoudakis N., Esteruelas M., Fort F., Canals J.M., Zamora F. (2011). Use of unripe grapes harvested during cluster thinning as a method for reducing alcohol content and pH of wine. Aust. J. Grape Wine Res..

[18] Sam F.E., Ma T., Liang Y., Qiang W., Atuna R.A., Amagloh F.K., Han S. (2021). Comparison between membrane and thermal dealcoholization methods: Their impact on the chemical parameters, volatile composition, and sensory characteristics of wines. Membranes.

[19] Herrera J., Bucchetti B., Sabbatini P., Comuzzo P., Zulini L., Vecchione A., Peterlunger E., Castellarin S. (2015). Effect of water deficit and severe shoot trimming on the composition of *Vitis vinifera* L. Merlot grapes and wines. Aust. J. Grape Wine Res..

[20] Caccavello G., Giaccone M., Scognamiglio P., Forlani M., Basile B. (2017). Influence of intensity of post-veraison defoliation or shoot trimming on vine physiology, yield components, berry and wine composition in Aglianico grapevines. Aust. J. Grape Wine Res..

[21] Gutiérrez-Gamboa G., Zheng W., De Toda M. (2021). Current viticultural techniques to mitigate the effects of global warming on grape and wine quality: A comprehensive review. Food Res. Int..

[22] Cincotta F., Verzera A., Prestia O., Tripodi G., Lechhab W., Sparacio A., Condurso C. (2022). Influence of leaf removal on grape, wine and aroma compounds of *Vitis vinifera* L. cv. Merlot under Mediterranean climate. Eur. Food Res. Technol..

[23] Zhang P., Wu X., Needs S., Liu D., Fuentes S., Howell K. (2017). The Influence of Apical and Basal Defoliation on the Canopy Structure and Biochemical Composition of *Vitis vinifera* cv. Shiraz Grapes and Wine. Front. Chem..

[24] Poni S., Casalini L., Bernizzoni F., Civardi S., Intrieri C. (2006). Effects of early defoliation on shoot photosynthesis, yield components, and grape composition. Am. J. Enol. Vitic..

[25] Sun Q., Sacks G.L., Lerch S.D., Heuvel J.E.V. (2012). Impact of shoot and cluster thinning on yield, fruit composition, and wine quality of Corot noir. Am. J. Enol. Vitic..

[26] Torres N., Martínez-Lüscher J., Porte E., Yu R., Kurtural S.K. (2021). Impacts of leaf removal and shoot thinning on cumulative daily light intensity and thermal time and their cascading effects of grapevine (*Vitis vinifera* L.) berry and wine chemistry in warm climates. Food Chem..

[27] Silveira J.M., Rombaldi C.V., del Aguila J.S., Gabbardo M., Cunha W.M.d. (2022). Agronomic and physicochemical parameters of must and wine as a function of changes in ‘Cabernet Sauvignon’ grapevine canopy. Acta Sci. Agron..

[28] Afonso S.M., Inês A., Vilela A. (2024). Bio-Dealcoholization of Wines: Can Yeast Make Lighter Wines?. Fermentation.

[29] Cataldo E., Salvi L., Paoli F., Fucile M., Mattii G.B. (2021). Effect of agronomic techniques on aroma composition of white grapevines: A review. Agronomy.

[30] Osrečak M., Karoglan M., Kozina B., Preiner D. (2015). Influence of leaf removal and reflective mulch on phenolic composition of white wines. OENO One.

[31] Meyers J.M., Sacks G.L., Heuvel J.E.V. (2013). Glycosylated aroma compound responses in ‘Riesling’ wine grapes to cluster exposure and vine yield. Hort. Technol..

[32] Mosetti D., Herrera J.C., Sabbatini P., Green A., Alberti G., Peterlunger E., Lisjak K., Castellarin S.D. (2016). Impact of leaf removal after berry set on fruit composition and bunch rot in ‘Sauvignon blanc’. J. Grapevine Res..

[33] Bureau S.M., Razungles A.J., Baumes R.L. (2000). The aroma of Muscat of Frontignan grapes: Effect of the light environment of vine or bunch on volatiles and glycoconjugates. J. Sci. Food Agric..

[34] Bubola M., Lukić I., Radeka S., Sivilotti P., Grozić K., Vanzo A., Bavčar D., Lisjak K. (2019). Enhancement of Istrian Malvasia wine aroma and hydroxycinnamate composition by hand and mechanical leaf removal. J. Sci. Food Agric..

[35] Palliotti A., Tombesi S., Frioni T., Silvestroni O., Bellincontro A., Poni S. (2014). Contenimento della produttività e dell’alcolicità potenziale di vini Sangiovese mediante posticipo della potatura invernale. Acta Italus Hortus—Atti del V Convegno Nazionale di Viticoltura, Foggia, Italy, 1–3 July 2014.

[36] Moran M.A., Sadras V.O., Petrie P.R. (2017). Late pruning and carry-over effects on phenology, yield components and berry traits in Shiraz. Aust. J. Grape Wine Res..

[37] Lanari V., Lattanzi T., Di Lena B., Palliotti A., Silvestroni O. (2019). Vite: Maturazione posticipata con la potatura tardiva. L’Informatore Agrario.

[38] Palliotti A., Frioni T., Tombesi S., Sabbatini P., Cruz-Castillo J., Lanari V., Silvestroni O., Gatti M., Poni S. (2017). Double-Pruning Grapevines as a Management Tool to Delay Berry Ripening and Control Yield. Am. J. Enol. Vitic..

[39] Martínez-Moreno A., Sanz F., Yeves A., Gil-Muñoz R., Martínez V., Intrigliolo D., Buesa I. (2019). Forcing bud growth by double-pruning as a technique to improve grape composition of *Vitis vinifera* L. cv. Tempranillo in a semi-arid Mediterranean climate. Sci. Hortic..

[40] Moran M., Bastian S., Petrie P., Sadras V. (2018). Late pruning impacts on chemical and sensory attributes of Shiraz wine. Aust. J. Grape Wine Res..

[41] Perin C., Verma P., Harari G., Suued Y., Harel M., Ferman-Mintz D., Drori E., Netzer Y., Fait A. (2023). Influence of late pruning practice on two red skin grapevine cultivars in a semi-desert climate. Front. Plant Sci..

[42] Cassano A., Conidi C., Drioli E. (2020). A comprehensive review of membrane distillation and osmotic distillation in agro-food applications. J. Membr. Sci. Res..

[43] Motta M. (2005). Introdução aos processos de separação por membranas. Material Didático da Disciplina de Processos Químicos de Tratamento de Efluentes.

[44] Lemperle T.J. (2010). Evaluation of Nanofiltration Membranes for the Production of a Dealcoholized Beverage at 0.5% vol. Master’s Thesis.

[45] Takács L., Vatai G., Korány K. (2007). Production of alcohol free wine by pervaporation. J. Food Eng..

[46] Vane L. (2005). A review of pervaporation for product recovery from biomass fermentation processes. J. Chem. Technol. Biotechnol.

[47] Sun X., Dang G., Ding X., Shen C., Liu G., Zuo C., Chen X., Xing W., Jin W. (2020). Production of alcohol-free wine and grape spirit by pervaporation membrane technology. Food Bioprod. Process..

[48] Labanda J., Vichi S., Llorens L., López-Tamames L. (2009). Membrane separation technology for the reduction of alcoholic degree of a white model wine. Food Sci. Technol..

[49] Ma T.Z., Sam F.E., Zhang B. (2022). Low-Alcohol and nonalcoholic wines: Production methods, compositional changes, and aroma improvement. Recent Advances in Grapes and Wine Production-New Perspectives for Quality Improvement.

[50] Diban N., Athes V., Bes M., Souchon I. (2008). Ethanol and aroma compounds transfer study for partial dealcoholization of wine using membrane contactor. J. Membr. Sci..

[51] Bes M., Aguera E., Athes V., Cadiere A., Cottereau P., Dequin S., Escudier J.L. (2010). Les différentes stratégies microbiologiques et technologiques de production de vin à teneur réduite en alcool. Rev. Anol. Tech. Vitivinic. Anol..

[52] Mangindaan D., Khoiruddin K., Wenten I.G. (2018). Beverage dealcoholization processes: Past, present, and future. Trends Food Sci. Technol..

[53] Banvolgyi S., Savaş Bahçeci K., Vatai G., Bekassy S., Bekassy-Molnar E. (2016). Partial dealcoholization of red wine by nanofiltration and its effect on anthocyanin and resveratrol levels. Food Sci. Technol. Int..

[54] Massot A., Mietton-Peuchot M., Peuchot C., Milisic V. (2008). Nanofiltration and reverse osmosis in winemaking. Desalination.

[55] Ivić I., Kopjar M., Jakobek L., Jukić V., Korbar S., Marić B., Mesić J., Pichler A. (2021). Influence of processing parameters on phenolic compounds and color of cabernet sauvignon red wine concentrates obtained by reverse osmosis and nanofiltration. Processes.

[56] Wright A.J., Pyle D.L. (1996). An investigation into the use of the Spinning Cone Column for in situ ethanol removal from yeast broth. Process Biochem..

[57] Grainger K., Tattersall H. (2005). Wine Production: Vine to Bottle.

[58] Filimon V.R., Tudose Sandu Ville Ș., Bora F.D., Tudor G., Filimon R., Nechita A., Damian D. (2020). Methods for Producing Low-Alcohol Wine I. Viticultural and Pre-Fermentation Strategies. https://www.uaiasi.ro/revista_horti/files/Nr1_2020/vol%2063_1_2020%20(15).pdf.

[59] Mermelstein N. (2000). Removing alcohol from wine. Food Technol. Mag..

[60] Pickering G.J. (2000). Low and Reduce-alcohol wine: A review. J. Wine Res..

[61] Lea A.G., Piggott J. (2003). Fermented Beverage Production.

[62] Motta S., Guaita M., Petrozziello M., Ciambotti A., Panero L., Solomita M., Bosso A. (2017). Comparison of the physicochemical and volatile composition of wine fractions obtained by two different dealcoholization techniques. Food Chem..

[63] Jackson R. (2008). Wine Science.

[64] Aguera E., Bes M., Roy A., Camarasa C., Sablayrolles J.M. (2010). Partial removal of ethanol during fermentation to obtain reduced-alcohol wines. Am. J. Enol. Vitic..

[65] Espejo F. (2021). Role of commercial enzymes in wine production: A critical review of recent research. J. Food Sci. Technol..

[66] Pickering G. (1997). The Production of Reduced-Alcohol Wine Using Glucose Oxidase. Ph.D. Thesis.

[67] Pickering G.J., Heatherbell D.A., Barnes M.F. (1998). Optimising Glucose Conversion in the Production of Reduced Alcohol Wine Using Glucose Oxidase. Food Res. Int..

[68] Pickering G.J., Heatherbell D.A., Barnes M.F. (1999). The Production of Reduced-Alcohol Wine Using Glucose Oxidase-Treated Juice. Part III. Sensory. Am. J. Enol. Vitic..

[69] Biyela B.N.E., du Toit W.J., Divol B., Malherbe D.F., van Rensburg P. (2009). The Production of Reduced-Alcohol Wines Using Gluzyme Mono^®^ 10.000 BG-Treated Grape Juice. S. Afr. J. Enol. Vitic..

[70] Motta J.F.G., Freitas B.C.B.D., Almeida A.F.D., Martins G.A.D.S., Borges S.V. (2023). Use of enzymes in the food industry: A review. Food Sci. Technol..

[71] Khatami S.H., Vakili O., Ahmadi N., Soltani Fard E., Mousavi P., Khalvati B., Maleksa-bet A., Savardashtaki A., Taheri-Anganeh M., Movahedpour A. (2022). Glucose oxidase: Applications, sources, and recombinant production. Biotechnol. Appl. Biochem..

[72] Röcker J., Schmitt M., Pasch L., Ebert K., Grossmann M. (2016). The use of glucose oxidase and catalase for the enzymatic reduction of the potential ethanol content in wine. Food Chem..

[73] Mangas R., González M.R., Martín P., Rodríguez-Nogales J.M. (2023). Impact of glucose oxidase treatment in high sugar and pH musts on volatile composition of white wines. LWT.

[74] Ferraro L., Fatichenti F., Ciani M. (2000). Pilot Scale Vinification Process Using Immobilized *Candida stellata* Cells and *Saccharomyces cerevisiae*. Process Biochem..

[75] Ciani M., Comitini F., Mannazzu I., Domizio P. (2010). Controlled Mixed Culture Fermentation: A New Perspective on the Use of Non-*Saccharomyces* Yeasts in Winemaking. FEMS Yeast Res..

[76] Comitini F., Gobbi M., Domizio P., Romani C., Lencioni L., Mannazzu I., Ciani M. (2011). Selected Non-Saccharomyces Wine Yeasts in Controlled Multistarter Fermentations with *Saccharomyces cerevisiae*. Food Microbiol..

[77] Magyar I., Tóth T. (2011). Comparative Evaluation of Some Oenological Properties in Wine Strains of *Candida stellata*, *Candida zemplinina*, *Saccharomyces uvarum* and *Saccharomyces cerevisiae*. Food Microbiol..

[78] Di Maio S., Genna G., Gandolfo V., Amore G., Ciaccio M., Oliva D. (2012). Presence of Candida zemplinina in Sicilian Musts and Selection of a Strain for Wine Mixed Fermentations. S. Afr. J. Enol. Vitic..

[79] Tofalo R., Schirone M., Torriani S., Rantsiou K., Cocolin L., Perpetuini G., Suzzi G. (2012). Diversity of *Candida zemplinina* Strains from Grapes and Italian Wines. Food Microbiol..

[80] Quirós M., Rojas V., Gonzalez R., Morales P. (2014). Selection of Non-Saccharomyces Yeast Strains for Reducing Alcohol Levels in Wine by Sugar Respiration. Int. J. Food Microbiol..

[81] Wang C., Mas A., Esteve-Zarzoso B. (2015). Interaction between *Hanseniaspora uvarum* and *Saccharomyces cerevisiae* during Alcoholic Fermentation. Int. J. Food Microbiol..

[82] Canonico L., Comitini F., Oro L., Ciani M. (2016). Sequential Fermentation with Selected Immobilized Non-*Saccharomyces* Yeast for Reduction of Ethanol Content in Wine. Front. Microbiol..

[83] Englezos V., Rantsiou K., Cravero F., Torchio F., Ortiz-julien A., Gerbi V., Rolle L., Co-colin L. (2016). *Starmerella bacillaris* and *Saccharomyces cerevisiae* Mixed Fermentations to Reduce Ethanol Content in Wine. Appl. Microbiol. Biotechnol..

[84] Varela C., Sengler F., Solomon M., Curtin C. (2016). Volatile flavour profile of reduced alcohol wines fermented with the non-conventional yeast species *Metschnikowia pulcherrima* and *Saccharomyces uvarum*. Food Chem..

[85] Ciani M., Morales P., Comitini F., Tronchoni J., Canonico L., Curiel J., Oro L., Rodrigues A., Gonzalez R. (2016). Non-conventional Yeast Species for Lowering Ethanol Content of Wines. Front. Microbiol..

[86] Lemos W.J.F., Nadai C., Tamara L., Sales V., Oliveira D., Dupas A., Matos D., Giacomini A., Corich V. (2019). Potential Use of Starmerella bacillaris as Fermentation Starter for the Production of Low-Alcohol Beverages Obtained from Unripe Grapes. Int. J. Food Microbiol..

[87] Milanovic V., Ciani M., Oro L., Comitini F. (2020). *Starmerella bombicola* Influences the Metabolism of *Saccharomyces cerevisiae* at Pyruvate Decarboxylase and Alcohol Dehydrogenase Level during Mixed Wine Fermentation. Microb. Cell Factories.

[88] Kutyna D., Varela C., Henschke P., Chambers P., Stanley G. (2010). Microbiological approaches to lowering ethanol concentration in wine. Trends Food Sci. Technol..

[89] Contreras A., Hidalgo C., Schmidt S., Henschke P., Curtin C., Varela C. (2015). The application of non-*Saccharomyces* yeast in fermentations with limited aeration as a strategy for the production of wine with reduced alcohol content. Int. J. Food Microbiol..

[90] Canonico L., Solomon M., Comitini F., Ciani M., Varela C. (2019). Volatile profile of reduced alcohol wines fermented with selected non-*Saccharomyces* yeasts under different aeration conditions. Food Microbiol..

[91] Jolly N., Mehlomakulu N., Nortje S., Beukes L., Hoff J., Booyse M., Erten H. (2022). Non-Saccharomyces yeast for lowering wine alcohol levels: Partial aeration versus standard conditions. FEMS Yeast Res..

[92] Klimczak K., Cioch-Skoneczny M., Ciosek A., Poreda A. (2024). Application of Non-Saccharomyces Yeast for the Production of Low-Alcohol Beer. Foods.

[93] Contreras A., Hidalgo C., Henschke P., Chambers P., Curtin C., Varela C. (2013). Evaluation of Non-Saccharomyces Yeasts for the Reduction of Alcohol Content in Wine. Appl. Environ. Microbiol..

[94] García M., Esteve-Zarzoso B., Cabellos J., Arroyo T. (2020). Sequential Non-*Saccharomyces* and *Saccharomyces cerevisiae* Fermentations to Reduce the Alcohol Content in Wine. Fermentation.

[95] Vilela A. (2020). Modulating Wine Pleasantness Throughout Wine-Yeast Co-Inoculation or Sequential Inoculation. Fermentation.

[96] Vicente J., Baran Y., Navascués E., Santos A., Calderón F., Marquina D., Rauhut D., Benito S. (2022). Biological management of acidity in wine industry: A review. Int. J. Food Microbiol..

[97] Ciani M., Comitini F. (2019). Use of Non-*Saccharomyces* Yeasts in Red Winemaking. Red Wine Technology.

[98] Hagman A., Piškur J. (2015). A Study on the Fundamental Mechanism and the Evolutionary Driving Forces behind Aerobic Fermentation in Yeast. PLoS ONE.

[99] Šoštarić N., Arslan A., Carvalho B., Plech M., Voordeckers K., Verstrepen K., Van Noort V. (2021). Integrated Multi-Omics Analysis of Mechanisms Underlying Yeast Ethanol Tolerance. J. Prot. Res..

[100] Vilela-Moura A., Schuller D., Mendes-Faia A., Côrte-Real M. (2008). Reduction of volatile acidity of wines by selected yeast strains. Appl. Microbiol. Biotechnol..

[101] Vilela A., Schuller D., Mendes-Faia A., Côrte-Real M. (2013). Reduction of volatile acidity of acidic wines by immobilized *Saccharomyces cerevisiae* cells. Appl. Microbiol. Biotechnol..

[102] Cuello R., Montero K., Mercado L., Combina M., Ciklic I. (2017). Construction of low-ethanol–wine yeasts through partial deletion of the *Saccharomyces cerevisiae* PDC2 gene. AMB Express.

[103] Puškaš V., Miljić U., Djuran J., Vučurović V. (2020). The aptitude of commercial yeast strains for lowering the ethanol content of wine. Food Sci Nut..

[104] Nogueira A., Mongruel C., Rosana D., Simões S. (2007). Effect of Biomass Reduction on the Fermentation of Cider. Braz. Arch. Biol. Technol..

[105] Nogueira A., Le Quéré J.M., Gestin P., Michel A., Wosiacki G., Drilleau J.F., Brew J.I. (2008). Slow Fermentation in French Cider Processing Due to Partial Biomass Reduction. J. Inst. Brew..

[106] Malfeito-Ferreira M. (2011). Yeasts and Wine Off-Flavours: A Technological Perspective. Ann. Microbiol..

[107] Guo F., Tang S., Wang R., Liu Q., Zhang J., Yang X., Li Y. (2012). Fermentation process of low-alcohol cider by biomass reduction. China Brew.

[108] Liszkowska W., Berlowska J. (2021). Yeast Fermentation at Low Temperatures: Adaptation to Changing Environmental Conditions and Formation of Volatile Compounds. Molecules.

[109] OECD (2021). Preventing Harmful Alcohol Use.

[110] de-la-Fuente-Blanco A., Arias-Pérez I., Escudero A., Sáenz-Navajas M.-P., Ferreira V. (2024). The relevant and complex role of ethanol in the sensory properties of model wines. OENO One.

[111] Kumar Y., Ricci A., Parpinello G.P., Versari A. (2024). Dealcoholized wine: A scoping review of volatile and non-volatile profiles, consumer perception, and health benefits. Food Bioproc. Technol..

[112] Branco Z., Baptista F., Paié-Ribeiro J., Gouvinhas I., Barros A.N. (2025). Impact of Winemaking Techniques on the Phenolic Composition and Antioxidant Properties of Touriga Nacional Wines. Molecules.

[113] Meillon S., Viala D., Medel M., Urbano C., Guillot G., Schlich P. (2010). Impact of Partial Alcohol Reduction in Syrah Wine on Perceived Complexity and Temporality of Sensations and Link with Preference. Food Qual. Prefer..

[114] Fedrizzi B., Nicolis E., Camin F., Bocca E., Carbognin C., Scholz M., Barbieri P., Finato F., Ferrarini R. (2014). Stable isotope ratios and aroma profile changes induced due to innovative wine dealcoholisation approaches. Food Bioprocess Technol..

[115] Longo R., Blackman J.W., Torley P.J., Rogiers S.Y., Schmidtke L.M. (2017). Changes in volatile composition and sensory attributes of wines during alcohol content reduction. J. Sci. Food Agric..

[116] Ma T., Sam F.E., Didi D.A., Atuna R.A., Amagloh F.K., Zhang B. (2022). Contribution of edible flowers on the aroma profile of dealcoholized pinot noir rose wine. LWT—Food Sci. Technol..

[117] Lisanti M.T., Gambuti A., Genovese A., Piombino P., Moio L. (2013). Partial dealcoholization of red wines by membrane contactor technique: Effect on sensory characteristics and volatile composition. Food Bioprocess Technol..

[118] Corona O., Liguori L., Albanese D., di Matteo M., Cinquanta L., Russo P. (2019). Quality and volatile compounds in red wine at different degrees of dealcoholization by membrane process. Eur. Food Res. Technol..

[119] Pham D.T., Stockdale V.J., Jeffery D.W., Tuke J., Wilkinson K.L. (2019). Investigating alcohol sweetspot phenomena in reduced alcohol red wines. Foods.

[120] Pham D.T., Ristic R., Stockdale V.J., Jeffery D.W., Tuke J., Wilkinson K. (2020). Influence of partial dealcoholization on the composition and sensory properties of Cabernet Sauvignon wines. Food Chem..

[121] Longo R., Blackman J.W., Antalick G., Torley P.J., Rogiers S.Y., Schmidtke L.M. (2018). Volatile and sensory profiling of Shiraz wine in response to alcohol management: Comparison of harvest timing versus technological approaches. Food Res. Int..

[122] Longo R., Blackman J.W., Antalick G., Torley P.J., Rogiers S.Y., Schmidtke L.M. (2018). A comparative study of partial dealcoholisation versus early harvest: Effects on wine volatile and sensory profiles. Food Chem..

[123] Puglisi C., Ristic R., Saint J., Wilkinson K. (2022). Evaluation of spinning cone column distillation as a strategy for remediation of smoke taint in juice and wine. Molecules.

[124] Ju Y., Xu X., Yu Y., Liu M., Wang W., Wu J., Liu B., Zhang Y., Fang Y. (2023). Effects of winemaking techniques on the phenolics, organic acids, and volatile compounds of Muscat wines. Food Biosci..

[125] Alises M.O., Sánchez-Palomo E., González Viñas M.A. (2024). Enhancing the Aroma of Dealcoholized La Mancha Tempranillo Rosé Wines with Their Aromatic Distillates. Beverages.

[126] Ivić I., Kopjar M., Buljeta I., Pichler D., Mesić J., Pichler A. (2022). Influence of Reverse Osmosis Process in Different Operating Conditions on Phenolic Profile and Antioxidant Activity of Conventional and Ecological Cabernet Sauvignon Red Wine. Membranes.

[127] Muñoz-González C., Martín-Álvarez P.J., Moreno-Arribas M.V., Pozo-Bayón M.Á. (2014). Impact of the nonvolatile wine matrix composition on the in vivo aroma release from wines. J. Agric. Food Chem..

[128] Muñoz-González C., Sémon E., Martín-Álvarez P., Guichard E., Moreno-Arribas M., Feron G., Pozo-Bayón M. (2015). Wine matrix composition affects temporal aroma release as measured by proton transfer reaction—Time-of-flight—Mass spectrometry. Aust. J. Grape Wine Res..

[129] Meillon S., Urbano C., Schlich P. (2009). Contribution of the Temporal Dominance of Sensations (TDS) Method to the Sensory Description of Subtle Differences in Partially Dealcoholized Red Wines. Food Qual. Prefer..

[130] Sam F.E., Ma T., Wang J., Liang Y., Sheng W., Li J., Jiang Y., Zhang B. (2023). Aroma improvement of dealcoholized Merlot red wine using edible flowers. Food Chem..

[131] Bucher T., Deroover K., Stockley C., Morata A., Loira I. (2019). Production and Marketing of Low-Alcohol Wine. Advances in Grape and Wine Biotechnology Advances in Grape and Wine Biotechnology.

[132] Bucher T., Frey E., Wilczynska M., Deroover K., Dohle S. (2020). Consumer perception and behavior related to low-alcohol wine: Do people overcompensate?. Public Health Nutr..

[133] Bisson L.F., Waterhouse A.L., Ebeler S.E., Walker M.A., Lapsley J.T. (2002). The present and future of the international wine industry. Nature.

[134] Naspetti S., Alberti F., Mozzon M., Zingaretti S., Zanoli R. (2019). Effect of information on consumer preferences and willingness-to-pay for sparkling mock wines. Br. Food J..

[135] D’hauteville F. (1994). Consumer Acceptance of Low Alcohol Wines. Int. J. Wine Mark..

[136] Day I., Deroover K., Kavanagh M., Beckett E., Akanbi T., Pirinen M., Bucher T. (2024). Australian consumer perception of non-alcoholic beer, white wine, red wine, and spirits. Int. J. Gastrom. Food Sci..

[137] Bruwer J., Jiranek V., Halstead L., Saliba A. (2014). Lower alcohol wines in the UK market: Some baseline consumer behaviour metrics. Br. Food J..

[138] Danner L., Ristic R., Johnson T.E., Meiselman H.L., Hoek A.C., Jeffery D.W., Bastian S.E.P. (2017). Context and wine quality effects on consumers’ mood, emotions, liking and willingness to pay for Australian Shiraz wines. Food Res. Int..

[139] van Leeuwen C., Destrac-Irvine A., Dubernet M., Duchêne E., Gowdy M., Marguerit E., Pieri P., Parlker A., de Reséguier L., Ollat N. (2019). An update on the impact of climate change in viticulture and potential adaptations. Agronomy.

[140] Haseeb S., Alexander B., Baranchuk A. (2017). Wine and cardiovascular health. Circulation.

[141] Jordão A.M., Vilela A., Cosme F. (2015). From Sugar of Grape to Alcohol of Wine: Sensorial Impact of Alcohol in Wine. Beverages.

[142] Rehm J., Neufeld M., Room R., Sornpaisarn B., Štelemėkas M., Swahn M.H., Lachenmeier D.W. (2022). The impact of alcohol taxation changes on unrecorded alcohol consumption: A review and recommendations. Int. J. Drug Policy.

[143] World Health Organization (2025). Harmful Use of Alcohol. https://www.who.int/health-topics/alcohol#tab=tab_1.

[144] Hay J.L., Kiviniemi M.T., Orom H., Waters E.A. (2023). Moving beyond the “Health Halo” of alcohol: What will it take to achieve population awareness of the cancer risks of alcohol?. Cancer Epidemiol. Biomark. Prev..

[145] Hrelia S., Di Renzo L., Bavaresco L., Bernardi E., Malaguti M., Giacosa A. (2022). Moderate wine consumption and health: A narrative review. Nutrients.

[146] Buljeta I., Pichler A., Šimunović J., Kopjar M. (2023). Beneficial effects of red wine polyphenols on human health: Comprehensive review. Curr. Issues Mol. Biol..

[147] Noguer M.A., Cerezo A.B., Donoso Navarro E., Garcia-Parrilla M.C. (2012). Intake of alcohol-free red wine modulates antioxidant enzyme activities in a human intervention study. Pharmacol. Res..

[148] Mihailovic-Stanojevic N., Savikin K., Zivkovic J., Zdunic G., Miloradovic Z., Ivanov M., Karanovic D., Vajic U.-J., Jovovic D., Grujic-Milanovic J. (2016). Moderate consumption of alcohol-free red wine provides more beneficial effects on systemic hemodynamics, lipid profile, and oxidative stress in spontaneously hypertensive rats than red wine. J. Funct. Foods.

[149] Castaldo L., Narváez A., Izzo L., Graziani G., Gaspari A., Di Minno G., Ritieni A. (2019). Red Wine Consumption and Cardiovascular Health. Molecules.

[150] Chiva-Blanch G., Urpi-Sarda M., Ros E., Arranz S., Valderas-Martínez P., Casas R., Estruch R. (2012). Dealcoholized red wine decreases systolic and diastolic blood pressure and increases plasma nitric oxide. Circ. Res..

[151] Chiva-Blanch G., Urpi-Sarda M., Ros E., Valderas-Martinez P., Casas R., Arranz S., Estruch R. (2013). Effects of red wine polyphenols and alcohol on glucose metabolism and the lipid profile: A randomized clinical trial. Clin. Nutr..

[152] Blalock D.V., Berlin S.A., Young J.R., Blakey S.M., Calhoun P.S., Dedert E.A. (2022). Effects of alcohol reduction interventions on blood pressure. Curr. Hypertens. Rep..

[153] Lamont K., Blackhurst D., Albertyn Z., Marais D., Lecour S. (2012). Lowering the alcohol content of red wine does not alter its cardioprotective properties. SAMJ S. Afr. Med. J..

[154] da Luz P.L., Serrano C.V., Chacra A.P., Monteiro H.P., Yoshida V.M., Furtado M., Ferreira S., Gutierrez P., Pileggi F. (1999). The effect of red wine on experimental atherosclerosis: Lipid-independent protection. Exp. Mol. Pathol..

[155] Mori T.A., Burke V., Zilkens R.R., Hodgson J.M., Beilin L.J., Puddey I.B. (2016). The effects of alcohol on ambulatory blood pressure and other cardiovascular risk factors in type 2 diabetes: A randomized intervention. J. Hypertens..

[156] Kabayama M., Akagi Y., Wada N., Higuchi A., Tamatani M., Tomita J., Kamide K. (2021). A randomized trial of home blood-pressure reduction by alcohol guidance during outpatient visits: OSAKE study. Am. J. Hypertens..

[157] Sabadashka M., Hertsyk D., Strugała-Danak P., Dudek A., Kanyuka O., Kucharska A.Z., Sybirna N. (2021). Anti-diabetic and antioxidant activities of red wine concentrate enriched with polyphenol compounds under experimental diabetes in rats. Antioxidants.

[158] Martin M.A., Goya L., Ramos S. (2017). Protective effects of tea, red wine and cocoa in diabetes. Evidences from human studies. Food Chem. Toxicol..

[159] Xia X., Sun B., Li W., Zhang X., Zhao Y. (2017). Anti-diabetic activity phenolic constituents from red wine against α-glucosidase and α-amylase. J. Food Process. Preserv..

[160] Belda I., Cueva C., Tamargo A., Ravarani C.N., Acedo A., Bartolomé B., Moreno-Arribas V. (2021). A multi-omics approach for understanding the effects of moderate wine consumption on human intestinal health. Food Funct..

[161] Queipo-Ortuño M.I., Boto-Ordóñez M., Murri M., Gomez-Zumaquero J.M., Clemente-Postigo M., Estruch R., Diaz F.D., Andrés-Lacueva C., Tinahones F.J. (2012). Influence of red wine polyphenols and ethanol on the gut microbiota ecology and biochemical biomarkers1234. Am. J. Clin. Nutr..

[162] Moreno-Indias I., Sánchez-Alcoholado L., Pérez-Martínez P., Andrés-Lacueva C., Cardona F., Tinahones F., Queipo-Ortuño M.I. (2016). Red wine polyphenols modulate fecal microbiota and reduce markers of the metabolic syndrome in obese patients. Food Funct..

[163] Nunes C., Figueiredo R., Laranjinha J., da Silva G.J. (2019). Intestinal cytotoxicity induced by *Escherichia coli* is fully prevented by red wine polyphenol extract: Mechanistic insights in epithelial cells. Chem. Biol. Interact..

[164] Lucerón-Lucas-Torres M., Cavero-Redondo I., Martínez-Vizcaíno V., Saz-Lara A., Pascual-Morena C., Álvarez-Bueno C. (2022). Association Between Wine Consumption and Cognitive Decline in Older People: A Systematic Review and Meta-Analysis of Longitudinal Studies. Front. Nutr..

[165] Gea A., Beunza J.J., Estruch R., Sánchez-Villegas A., Salas-Salvadó J., Buil-Cosiales P., Gómez-Gracia E., Covas M.-I., Corella D., Fiol M. (2013). Alcohol intake, wine consumption and the development of depression: The PREDIMED study. BMC Med..

[166] Rogers P., Giannasio M. On Wine and Food, Together. Drinks and Restaurants. Proceedings of the 2020 Dublin Gastronomy Symposium.

[167] Nikolaou A., Santarmaki V., Mitropoulou G., Sgouros G., Kourkoutas Y. (2023). Novel Low-Alcohol Sangria-Type Wine Products with Immobilized Kefir Cultures and Essential Oils. Microbiol. Res..

[168] Kustos M., Heymann H., Jeffery D., Goodman S., Bastian S. (2020). Intertwined: What makes food and wine pairings appropriate?. Food Res. Int..

[169] Kustos M., Goodman S., Jeffery D., Bastian S. (2021). Appropriate food and wine pairings and wine provenance information: Potential tools for developing memorable dining experiences. Food Qual. Pref..

[170] Serra M., António N., Henriques C., Afonso C. (2021). Promoting Sustainability through Regional Food and Wine Pairing. Sustainability.

[171] Takahashi T., Nakano K., Yamashita M., Yamazaki H., Fushiki T. (2021). Pairing of white wine made with shade-grown grapes and Japanese cuisine. NPJ Sci. Food.

[172] Wang S. (2017). When Chinese cuisine meets western wine. Int. J. Gastron. Food Sci..

[173] Kim C., Lecat B. (2017). An Exploratory Study to Develop Korean Food and Wine Pairing Criteria. Beverages.

[174] Cosme F., Pinto T., Vilela A. (2017). Oenology in the Kitchen: The Sensory Experience Offered by Culinary Dishes Cooked with Alcoholic Drinks, Grapes and Grape Leaves. Beverages.

[175] Spence C. (2020). Food and beverage flavour pairing: A critical review of the literature. Food Res. Int..

[176] Gawrysiak Z., Żywot A., Ławrynowicz A. (2024). WineGraph: A Graph Representation for Food-Wine Pairing. International Conference on Neural-Symbolic Learning and Reasoning.

[177] Eschevins A., Giboreau A., Julien P., Dacremont C. (2019). From expert knowledge and sensory science to a general model of food and beverage pairing with wine and beer. Int. J. Gastron. Food Sci..

[178] Harrington R.J. (2007). Food and Wine Pairing.

[179] Harrington R.J. (2008). The Wine and Food Pairing Process. J. Culin. Sci. Technol..

[180] Joachim D., Schloss A. (2017). Alcohol’s Role in Cooking. Fine Cook.

[181] López R., Wen Y., Ferreira V. (2024). The remarkable effects of the non-volatile matrix of wine on the release of volatile compounds evaluated by analyzing their release to the headspaces. OENO One.

[182] Villamor R., Ross C. (2013). Wine matrix compounds affect perception of wine aromas. Ann. Rev. Food Sci. Technol..

[183] Sáenz-Navajas M., Campo E., Culleré L., Fernández-Zurbano P., Valentin D., Ferreira V. (2010). Effects of the non-volatile matrix on the aroma perception of wine. J. Agric. Food Chem..

[184] Robinson A., Ebeler S., Heymann H., Boss P., Solomon P., Trengove R. (2009). Interactions between wine volatile compounds and grape and wine matrix components influence aroma compound headspace partitioning. J. Agric. Food Chem..

[185] (1977). Sensory Analysis—Apparatus—Wine-Tasting Glass.

[186] Pittari E., Moio L., Piombino P. (2021). Interactions between Polyphenols and Volatile Compounds in Wine: A Literature Review on Physicochemical and Sensory Insights. Appl. Sci..

[187] González-Muñoz B., Garrido-Vargas F., Pavez C., Osorio F., Chen J., Bordeu E., O’Brien J., Brossard N. (2021). Wine Astringency: More Than Just Tannin-Protein Interactions. J. Sci. Food Agric..

[188] Medel-Marabolí M., Romero J., Obreque-Slier E., Contreras A., Peña-Neira Á. (2017). Effect of a commercial tannin on the sensorial temporality of astringency. Food Res. Int..

[189] Varela C., Barker A., Tran T., Borneman A., Curtin C. (2017). Sensory profile and volatile aroma composition of reduced alcohol Merlot wines fermented with *Metschnikowia pulcherrima* and *Saccharomyces uvarum*. Int. J. Food Microbiol..

[190] Vilela A., Inês A., Cosme F. (2016). Is wine savory? Umami taste in wine. SDRP J. Food Sci. Technol..

[191] Vilela A., Jordão A., Cosme F. (2016). Wine phenolics: Looking for a smooth mouthfeel. SDRP J. Food Sci. Technol..

[192] Snitkjær P., Risbo J., Skibsted L.H., Ebeler S., Heymann H., Harmon K., Frøst M.B. (2011). Beef stock reduction with red wine—Effects of preparation method and wine characteristics. Food Chem..

